# Detachment‐Induced FAK‐STAT3‐NNMT Inhibits CTCs Anoikis to Promote Breast Cancer Metastasis by Enhancing Fatty Acid Oxidation

**DOI:** 10.1002/advs.202522837

**Published:** 2026-03-12

**Authors:** Qingchao Tong, Yilei Ma, Yuzhen Gao, Lin Zhou, Luping Lou, Qiang Fang, Sining Fang, Ru Zhao, Jin Zeng, Jun Yang, Guoli Li, Xinyou Xie, Yanzhong Wang, Jun Zhang

**Affiliations:** ^1^ Department of Clinical Laboratory Sir Run Run Shaw Hospital Zhejiang University School of Medicine Hangzhou Zhejiang P. R. China; ^2^ Key Laboratory of Precision Medicine in Diagnosis and Monitoring Research of Zhejiang Province Hangzhou Zhejiang P. R. China

**Keywords:** anoikis, breast cancer, CTCs, FAO, NNMT

## Abstract

Breast cancer metastasis claims the majority of breast cancer‐related deaths. Anoikis resistance is a key prerequisite for CTCs survival and metastasis. Previous studies have demonstrated that Nicotinamide N‐methyltransferase (NNMT) plays a crucial role in cancer metastasis and apoptosis resistance. However, whether NNMT participates in breast cancer CTCs anoikis remains unexplored. In this study, the upregulation of NNMT was observed in CTCs from breast cancer patients and mouse CTCs models. NNMT in detached breast cancer cells is induced by FAK‐STAT3 axis and resists anoikis through FAO activation, promoting CTCs survival. Mechanistically, NNMT promotes the expression of CPT1A and CD36 by suppressing PP2A methylation to enhance FAO. Furthermore, NNMT‐induced FAO accelerates ROS clearance by maintaining NADP+/NADPH balance. In vivo experiments show that NNMT‐knockdown, NNMT inhibitors and FAO inhibitors can all reduce lung metastases formation, suggesting that targeting NNMT‐FAO suppresses the metastatic potential of breast cancer. Our study revealed that the upregulation of NNMT is induced by FAK‐STAT3 axis, which contributes to CTCs anoikis resistance in breast cancer by activating FAO. Targeting NNMT may provide new therapeutic targets for metastatic breast cancer.

## Graphical abstract

The FAK‐STAT3‐NNMT axis drives anoikis resistance in circulating tumor cells by reprogramming fatty acid oxidation. Targeting this metabolic vulnerability suppresses metastasis, untangling a key mechanism of breast cancer progression and revealing NNMT as a promising therapeutic target. This study unravels how CTCs survive in circulation, presenting a metabolic target for therapeutic intervention.

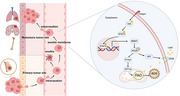

## 1 Introduction

Breast cancer now accounts for close to one in four cancer cases (23.8%) and one in six cancer deaths (15.4%) in women worldwide [1]. It's reported that 20%–30% of breast cancer patients develop metastases after diagnosis and that approximately 90% of cancer‐related deaths can be attributed to metastasis [2, 3]. Cancer metastasis is a complicated multistep process that can be broadly categorized into five distinct stages: detachment from the primary tumor, invasion into the peripheral circulation, survival in the bloodstream, extravasation from the circulation, and colonization of the metastatic site [4]. Circulating tumor cells (CTCs) refer to tumor cells that are detached from the primary tumor and survive in the circulatory system, which play a significant role in the process of tumor metastasis. As CTCs detach from the primary tumor and enter circulation, they encounter numerous challenges to survive and establish new tumors in distant organs [5]. Although up to 1 × 10^6^ cancer cells per gram of tumor tissue are estimated to be shed into circulation, the metastasis efficiency of CTCs is extremely low, approximately 0.01% [6].

Circulating tumor cells encounter many survival challenges during circulation, including anoikis, fluid shear stress, oxidative damage, and immune surveillance [7]. During this process, CTCs employ various mechanisms to survive, thereby promoting metastasis [8]. Due to detachment from the extracellular matrix (ECM), CTCs typically activate several molecular pathways, including Focal Adhesion Kinase(FAK), Src, and AMP‐activated protein kinase(AMPK) [9, 10], which are essential for the survival of CTCs. [11, 12]. These alterations in signaling pathways may induce changes in the metabolic behavior of tumor cells. For instance, FAK was reported to participate in glucose, lipid, and glutamine metastasis to promote cancer development, while AMPK can induce a metabolic shift from aerobic glycolysis to fatty acid oxidation [13]. Recent research has revealed that FAO provides an important survival advantage for cancer development by supplying Nicotinamide adenine dinucleotide(NADH), Nicotinamide adenine dinucleotide phosphate hydrogen (NADPH), and ATP [14]. In breast cancer, Fatty acid oxidation(FAO) has been found to contribute to cancer development, such as proliferation, cancer stemness, and chemoresistance [15], particularly under metabolic stress conditions or nutrient restriction [16]. However, the underlying mechanism by which FAO is activated in detached breast cancer cells still requires further research.

The cancer metabolizing enzyme nicotinamide N‐methyltransferase (NNMT) catalyzes the transfer of a methyl group from S‐adenosyl‐L‐methionine (SAM) to nicotinamide (NA), generating S‐adenosylhomocysteine (SAH) and 1‐methyl‐nicotinamide (1‐MNA). The Upregulation of NNMT has been observed in various types of cancer, including prostate cancer [17], colon cancer [18], and breast cancer [19], in both cancerous and stromal cells [20], which was reported to promote cancer cell metastasis [21], proliferation [22], and chemotherapy resistance [23]. Several studies have reported that NNMT can consume methyl donor SAM to competitively inhibit the methylation of various molecules, including histones and non‐histone proteins like PP2A [24], and NNMT has been implicated in promoting cancer metastasis through this mechanism [25]. Our previous study has demonstrated that NNMT positively correlates with lymph node metastasis and distant metastasis in breast cancer patients. It also plays a role in resisting apoptosis in breast cancer [26]. This study preliminarily revealed that NNMT was highly expressed in breast cancer CTCs and upregulated in detached breast cancer cells. Therefore, we hypothesize that NNMT may play a role in breast cancer CTCs formation to promote distant metastasis.

In the present study, we found that the upregulation of NNMT plays a crucial role in breast cancer anoikis resistance to promote CTCs survival. The upregulation of NNMT in detached breast cancer cells is mediated by the FAK‐STAT3 axis. And NNMT induces anoikis resistance in breast cancer by upregulating CPT1A, thereby enhancing fatty acid oxidation. The upregulation of CPT1A is achieved through the inhibition of PP2A methylation. Moreover, inhibition of the NNMT‐FAO axis using etomoxir or an NNMT inhibitor exhibited a significant inhibitory effect on cancer metastasis, as compared with the control group, indicating the possibility of targeting the NNMT‐FAO axis as a potential therapeutic strategy for metastatic breast cancer. Collectively, our study demonstrated that the FAK‐STAT3‐NNMT axis could promote CTCs survival to enhance breast cancer metastasis by activating FAO to resist anoikis.

## 2 Results

### 2.1 NNMT Expression is Up‐Regulated in Breast Cancer CTCs

**FIGURE 1:**
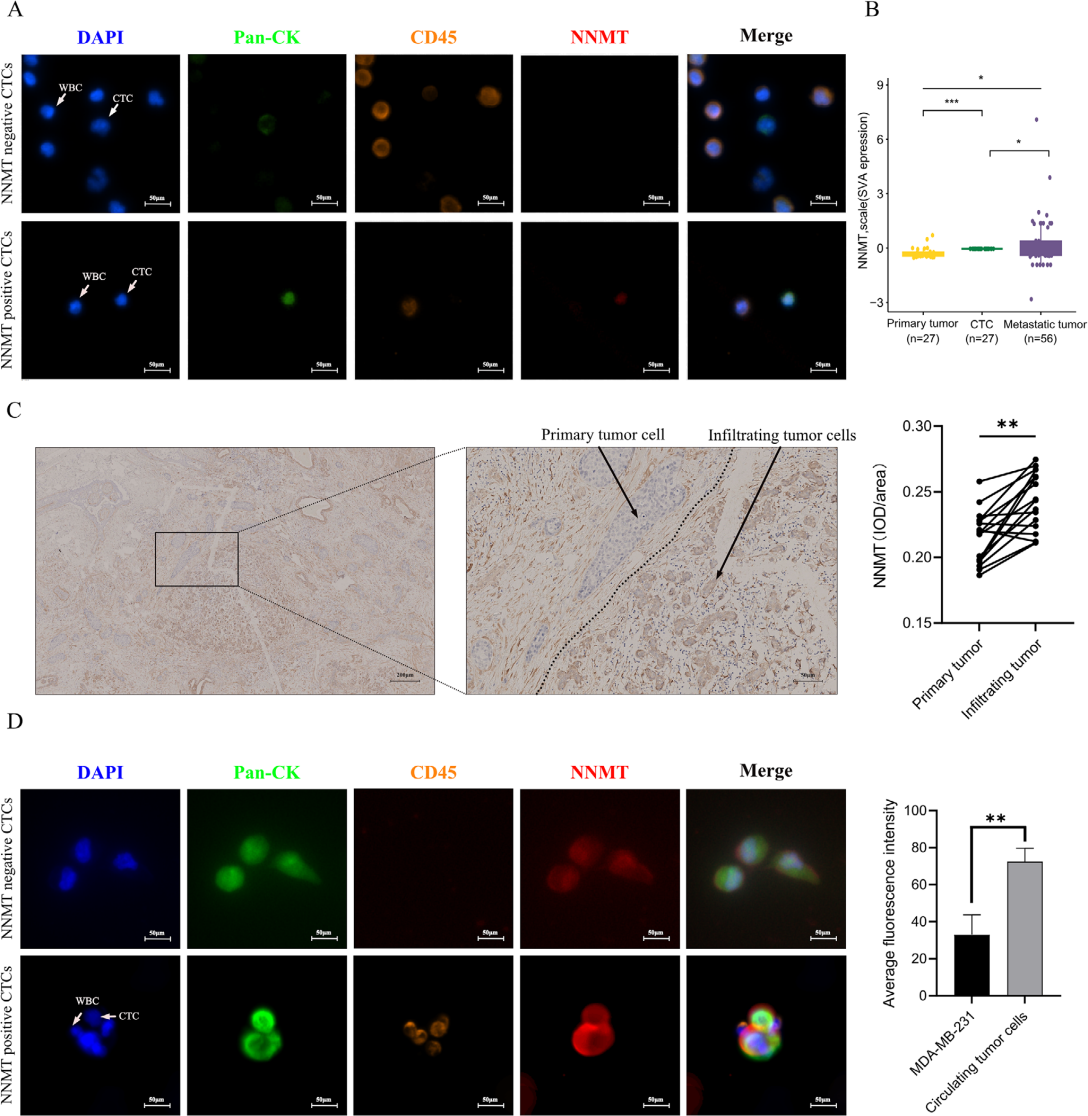
NNMT is upregulated in detached breast cancer cells. (A) Representative result of NNMT expression in breast cancer patient CTCs. Scale bars, 50 µm. (B) Representative result of NNMT expression in primary lesions, CTCs, and metastatic lesions. Data expressed as mean ± SEM; ^*^ *p* < 0.05, ^***^ *p* < 0.001, by one‐way ANOVA. (C) Representative images of NNMT immunohistochemical analysis and statistical analysis of the integrated optical density of NNMT in the invading edge and tumor of 17 breast cancer patients. The “primary tumor” and “infiltrating edge” regions were selected based on morphological features by an experienced pathologist according to Guidelines for breast cancer diagnosis and treatment by China Anti‐cancer Association (2024 edition). Data were presented as mean ± SEM ^**^ *p* < 0.01. D: Representative result of NNMT expression between MDA‐MB‐231 and isolated CTC cells by Immunofluorescence assay (*n* = 5 for CTCs group). Scale bars, 50 µm. Human CTCs were defined as Pan‐CK^+^, CD45^−^ and DAPI^+^. CK^−^, CD45^+^ and DAPI^+^ cells indicate white blood cells. Data were presented as mean ± SEM. ^**^ *p* < 0.01.

Our previous studies have reported the higher expression of NNMT in breast cancers [25] and correlated with breast cancer metastasis [25], apoptosis resistance [19], and progression [27]. Therefore, we hypothesized that NNMT participated in CTCs formation and survival to promote tumor metastasis. First, we collected blood samples from 45 patients with newly diagnosed breast cancer and 17 primary breast cancer tissues with invading edges. However, only 10 blood samples contained CTCs due to the limited amount of blood samples and the early stage of the tumor, but NNMT‐positive CTCs were detected in 9 patients (Figure 1A; Figure S1A). The proportion of breast cancer patients with NNMT^+^ CTCs in all patients with CTCs was as high as 90%. Among the 45 breast cancer patients, distant metastases were found in only one case, who had NNMT^+^ CTCs. The lymph node metastases were observed in 4 out of 9 patients with NNMT^+^ CTCs, while the patient with NNMT^−^ CTCs did not exhibit any lymph node metastases (Tables S1 and S2). NNMT expression was detected in a subset of CTCs, suggesting a possible association with early dissemination or survival in circulation. Then, we analyzed sequencing data from three sources of breast cancer in two cohorts (GSE209998 and GSE113890): primary tumor, peripheral CTCs, and metastatic tumor. Estimation of surrogate variables using the sva package allowed for effective batch correction and integration of gene expression data across the two datasets, as shown in Figure S1B, which revealed that the expression of NNMT in CTCs and metastasis tumor were significantly higher than that of in situ breast cancer samples (Figure 1B). However, NNMT expression of metastasis tumor was variable across different metastatic lesions, and in some sites exceeded that of CTCs. As shown in Figure 1C, NNMT staining intensity was observed to be higher at the infiltrating edge compared to the primary site from the same patient, as supported by quantitative analysis across 17 samples. This indicates that tumor cells at the invasive front exhibit higher NNMT expression than those in the primary tumor region. Besides, we also established the CTCs model by injecting MDA‐MB‐231 breast cancer cells into the mammary fat pads of SCID mice, and an immunofluorescence assay confirmed that NNMT expression was, indeed, increased in isolated CTCs compared to implanted MDA‐MB‐231 (Figure 1D). Moreover, to mimic the non‐adherent conditions encountered by CTCs, we established a suspension culture model using MDA‐MB‐231 breast cancer cells under ultra‐low attachment conditions and performed RNA‐seq, revealing that NNMT expression in detached breast cancer cells was 2.74‐fold higher than that in attached breast cancer cells (Table S4), and the results of WB and QPCR also showed that NNMT expression was significantly higher in detached breast cancer cells compared to that in attached cells (Figure S1C,D). To sum up, these results confirmed that NNMT was upregulated in breast cancer cells during detachment.

### 2.2 The Upregulated NNMT in Detached Breast Cancer Cells is Induced by the FAK‐STAT3 Axis

**FIGURE 2:**
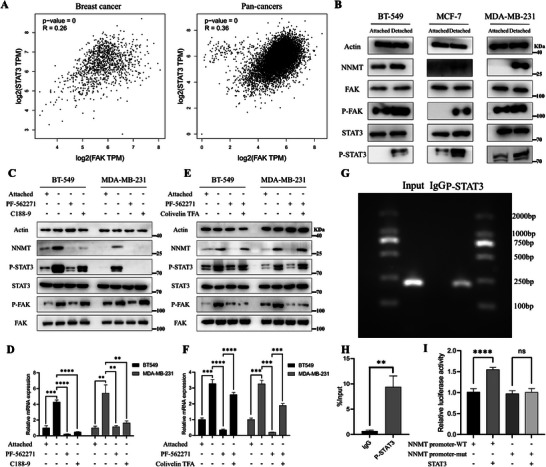
Detachment‐induced NNMT is upregulated by the FAK‐STAT3 axis. (A) Representative image of the positive correlation between FAK and STAT3 in breast cancer and pan‐cancer according to the GEPIA 2 database analysis. (B) Representative results of STAT3, P‐STAT3, FAK, and P‐FAK proteins by WB in the detached BT‐549, MCF‐7, and MDA‐MB‐231 cells. (C,D) The protein and mRNA levels of NNMT and P‐STAT3 after treating detached cells with FAK inhibitor PF‐562271 (10 µm) and P‐STAT3 inhibitor C188‐9 (10 µm) for 48 h by WB and QPCR. ^**^ *p* < 0.01, ^***^ *p* < 0.001, ^****^ *p* < 0.0001. (E,F) The protein and mRNA levels of NNMT and P‐STAT3 after treating detached cells with FAK inhibitor PF‐562271 (10 µm for 48 h) and P‐STAT3 activator (1 µm for 24 h) by WB and QPCR. ^**^ *p* < 0.01, ^***^ *p* < 0.001, ^****^ *p* < 0.0001. (G,H) ChIP assay using P‐STAT3 antibodies in MDA‐MB‐231 cells cultured under suspension conditions was performed, followed by PCR and qPCR, which showed P‐STAT3 enrichment at the NNMT promoter vs. IgG control. ^**^ *p* < 0.01. I: Dual‐luciferase reporter assays showed that co‐transfection with the full‐length NNMT promoter and a STAT3‐expressing plasmid significantly enhanced luciferase activity, whereas this enhancement was abolished when a truncated NNMT promoter was used. Data were presented as mean ± SEM.^****^ *p* < 0.0001.

When cancer cells detach from ECM, integrins will recruit and phosphorylate FAK, which subsequently activates downstream signaling molecules [28]. Since STAT3 was one upstream regulator of NNMT [29] and it has been reported that STAT3 can be activated by the Integrin‐FAK pathway [30], we investigated whether the known FAK–STAT3–NNMT signaling axis is activated in detached breast cancer cells and contributes to NNMT transcriptional upregulation under anchorage‐independent conditions. According to the GEPIA 2 database. Correlation analysis has indicated a positive correlation between the expression of STAT3 and FAK in both breast cancer and pan‐cancers (Figure 2A), and the results of the BioGRID 4.4 database also suggested an interaction between STAT3 and FAK (Figure S2A). Then, we have further explored the RPPA dataset for breast cancer, focusing on phosphorylated FAK and phosphorylated STAT3. Preliminary analysis shows positive correlation between p‐FAK and p‐STAT3 levels (Figure S2B), supporting our cell line‐based findings. The STRING database also showed that STAT3 and FAK exhibited potential interaction (Figure S2C). Grayscale quantification of WB bands showed the elevated expression levels of both P‐FAK and P‐STAT3 in detached breast cancer cells, suggesting the activation of FAK and STAT3 in detached breast cancer cells (Figure 2B; Figure S2D). To verify our hypothesis, we treated detached breast cancer cells with FAK inhibitor PF562271 and P‐STAT3 inhibitor C188‐9. Both qPCR and WB results showed that NNMT expression in detached cells decreased by inhibition of FAK or STAT3 (Figure 2C,D). Genetic suppression of FAK/STAT3 by siRNA significantly downregulates NNMT in suspended breast cancer cells (Figure S2E). Moreover, NNMT expression could be rescued by STAT3 activator Colivelin TFA (Figure 2E,F). These results indicate that upregulation of NNMT in detached breast cancer cells was induced by the FAK‐STAT3 axis. To investigate whether STAT3 directly regulates NNMT transcription, we conducted in silico analysis of the NNMT promoter region using the JASPAR database to identify putative STAT3‐binding motif(Figure S2F) and performed chromatin immunoprecipitation (ChIP) using P‐STAT3 antibodies in MDA‐MB‐231 cells cultured under suspension conditions (Figure 2G,H). In parallel, we also performed comprehensive dual‐luciferase reporter assays, which revealed that co‐transfection of the full‐length NNMT promoter reporter with STAT3 resulted in strong luciferase activation. Most critically, the truncated promoter plasmid group exhibited a significant reduction in luciferase activity compared to the full‐length promoter upon STAT3 activation. (Figure 2I). These results probe the binding of P‐STAT3 to the NNMT promoter in detached breast cancer cells. In summary, we have identified NNMT as a critical and functional downstream target of detachment‐induced FAK‐STAT3 signaling in breast cancer cells.

### 2.3 NNMT Induces Breast Cancer Anoikis Resistance to Increase the Number of CTCs

**FIGURE 3:**
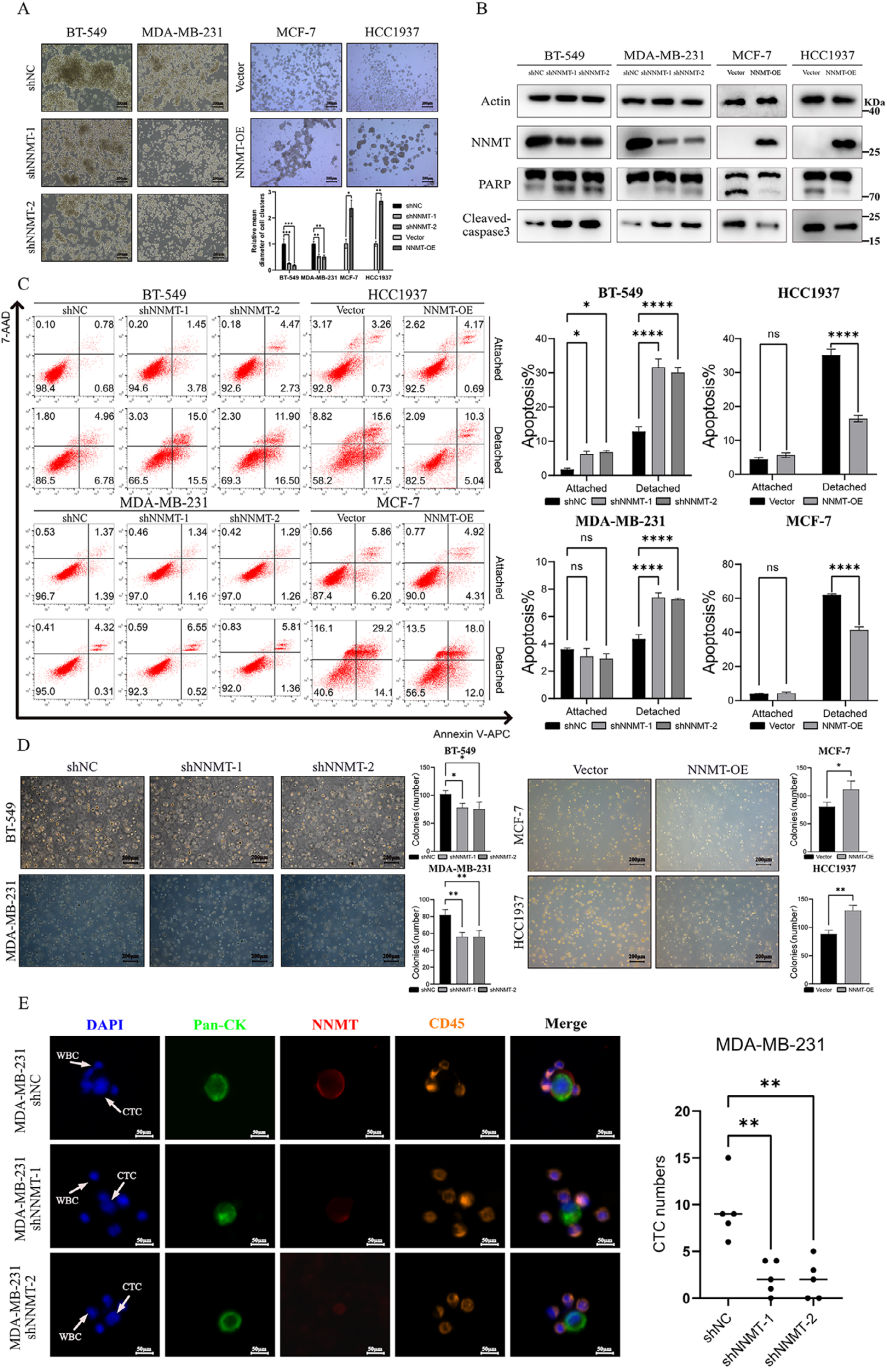
NNMT promotes anoikis resistance in breast cancer cells. (A) Cell morphology after detachment for 48 h. Scale bar, 200 µm. (B) Representative results of anoikis levels of BT‐549, MCF‐7, HCC1937, and MDA‐MB‐231 cell lines by Annexin V/7‐AAD staining after cultured on 1% agar plates for 48 h. ^**^ *p* < 0.01, ^***^ *p* < 0.001, ^****^ *p* < 0.0001. (C) WB analysis showed that cleaved‐PARP and cleaved‐caspase3 increased in NNMT‐knockdown BT‐549 and MDA‐MB‐231 cells, while decreased in NNMT‐overexpression MCF‐7 and HCC1937 cells. (D) Representative result of soft agar assay of BT‐549, MCF‐7, MDA‐MB‐231, and HCC1937. (E) Representative result of isolated CTCs from the control and NNMT‐knockdown group. Scale bars, 50 µm. CTCs in the mouse model were defined as Pan‐CK^+^, CD45^−^ and DAPI^+^. CD45^+^, CK^−^ and DAPI^+^ cells indicate white blood cells (*n* = 5 for each group). Scale bar, 50 µm. Data were presented as mean ± SEM. ^*^ *p* < 0.05, ^**^ *p* < 0.01.

CTCs are a key step in the process of tumor metastasis, and anoikis resistance is a key prerequisite for CTCs survival. To explore the function of NNMT in breast cancer anoikis resistance, we established NNMT‐knockdown MDA‐MB‐231 and BT‐549 breast cancer cell lines via lentiviral transfection and NNMT‐overexpression MCF‐7 and HCC1937 cell models. The effect of NNMT knockdown or overexpression was confirmed at mRNA levels (Figure S3A). After culturing these 4 cell lines in suspension for 48 h, we observed that NNMT overexpression increased cell aggregation while knockdown decreased it (Figure 3A), indicating the potential role of NNMT in promoting anoikis resistance in detached breast cancer cells. WB analysis of anoikis markers, PARP and cleaved‐caspase3, showed that NNMT‐knockdown cells showed a higher proportion of anoikis, and NNMT‐overexpression cells showed a lower proportion of anoikis (Figure 3B). Anoikis assay revealed that NNMT knockdown or overexpression resulted in only minor changes in apoptosis proportion upon attachment (Figure 3C). In contrast, when cancer cells detached from the culture plate, NNMT knockdown significantly increased the anoikis proportion in BT‐549 (from 8.01% to 27.8%) and MDA‐MB‐231 (from 3.08% to 6.4%), while NNMT overexpression protected MCF‐7 and HCC1937 from anoikis (respectively from 43.3% to 30%, 33.1% to 15.34%) from anoikis compared to control group (Figure 3C). Interestingly, the difference in the proportion of anoikis among these 4 cell lines correlated with their level of natural NNMT expression (Figure S3B) since cell lines with high NNMT expression, such as MDA‐MB‐231 and BT‐549, exhibited a low proportion of anoikis while cell lines with low NNMT‐expression, such as MCF‐7 and HCC1937, exhibited a high proportion of anoikis. Results of soft agar assay showed that NNMT‐knockdown in BT‐549 and MDA‐MB‐231 cells significantly decreased the number of cell colonies, while NNMT‐overexpression in MCF‐7 and HCC1937 cells increased the number of cell colonies, which both consisted with our hypothesis (Figure 3D). To further validate these findings, we re‐expressed NNMT by transfecting an NNMT plasmid into MDA‐MB‐231 NNMT‐knockdown cells. A subsequent anoikis assay revealed that the reintroduction of NNMT significantly suppressed anoikis (Figure S4), thereby confirming our conclusion. Then, we constructed a CTCs model with MDA‐MB‐231 cells, and the results showed that the number of CTCs was significantly reduced in the NNMT knockdown group compared with the control group (Figure 3E), suggesting the crucial role of NNMT in breast cancer CTCs anoikis resistance. These findings demonstrated that NNMT could induce breast cancer cell anoikis resistance.

### 2.4 NNMT Promoted FAO in Attached and Detached Breast Cancer Cells

**FIGURE 4:**
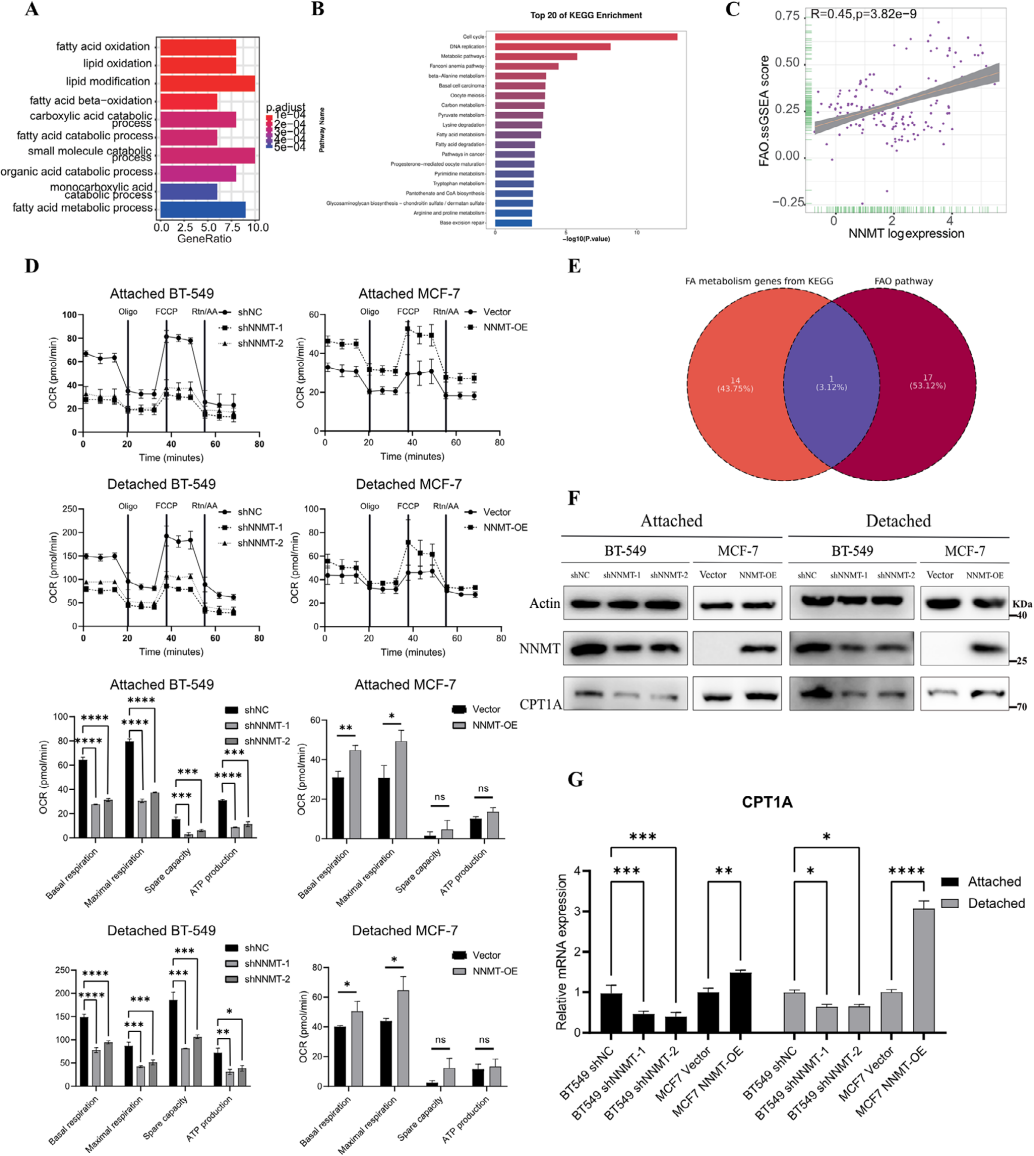
NNMT promoted FAO in attached and detached breast cancer cells. (A) The top 10 NNMT‐related pathways among the CTC compared primary lesions from combined cohorts (GSE9893). (B) The results of the top 20 enrichment pathways by KEGG pathway enrichment analysis among the detached MDA‐MB‐231 compared to the attached MDA‐MB‐231. (C) Relationship between the mRNA expression of the FAO pathway and NNMT in breast cancer patients from combined cohorts. (D) Measurements of FAO rate in attached and detached BT‐549 MCF‐7 cell models using the Seahorse XF96e extracellular flux analyzer. ^*^ *p* < 0.05, ^**^ *p* < 0.01, ^****^ *p* < 0.0001. (E) The results of the Venn diagram between the fatty acid metabolism from KEGG pathway enrichment analysis and the functional gene of the FAO pathway. (F,G) WB and QPCR analysis showed CPT1A was upregulated by NNMT in BT‐549 and MCF‐7, whether detached or not. Data were presented as mean ± SEM. ^*^ *p* < 0.05, ^**^ *p* < 0.01, ^****^ *p* < 0.0001.

To investigate the mechanism of breast cancer resistance to anoikis, we preliminarily analyzed differential gene expression in CTCs and primary breast cancer tissue (Table S5). Through enrichment analysis, we found FAO to be among the top 10 dysregulated activation pathways of CTCs (Figure 4A), indicating its potential role in breast cancer during detachment. Enrichment result of the P‐adjust<0.001 pathway was listed in Table S6. Besides, according to the RNA‐seq results, we conducted KEGG pathway analysis and discovered that a considerable number of differentially expressed genes were enriched in the fatty acid metabolism pathway of detached MDA‐MB‐231 cells (Figure 4B). Then, we observed a positive correlation (r = 0.45, *p*<0.05) between FAO and NNMT in the combined breast cancer cohort (Figure 4C). These results indicate that NNMT may impact fatty acid utilization. To directly investigate the function of NNMT in FAO rate, we performed the FAO assay with Seahorse XF96e Extracellular Flux Analyzer. After starvation and exogenous palmitate treatment, basal respiration and maximal respiration both decreased in NNMT‐knockdown BT‐549 cells compared to the control group. Conversely, maximal respiration increased notably in NNMT‐overexpressing MCF‐7 cells relative to the control group (Figure 4D). Further analysis identified CPT1A, the key FAO rate‐limiting enzyme, as being present in both the fatty acid metabolism pathway from KEGG pathway analysis and the FAO pathway geneset (Figure 4E). This finding suggested that NNMT may regulate CPT1A to enhance FAO. To validate this hypothesis, we assessed the expression of CPT1A in attached and detached breast cancer cells. Our results highlighted that the protein and mRNA levels of CPT1A were higher in NNMT‐overexpressing MCF‐7 cells and lower in NNMT‐knockdown BT‐549 cells under both detachment and attachment conditions (Figure 4F,G). Similar results were observed in MDA‐MB‐231 cells (Figure S5), indicating that NNMT can enhance fatty acid oxidation (FAO) in both detached and attached breast cancer cells. Additionally, other genes involved in fatty acid metabolism, such as CD36 [31, 32] and ACC [33], have also been reported to be able to modulate FAO. Therefore, we investigated the role of NNMT in fatty acid uptake and synthesis by examining its role in the regulation of CD36, a key gene involved in fatty acid uptake, and ACC, which is crucial for fatty acid synthesis. The results of WB and QPCR showed that NNMT increased CD36 expression rather than ACC in both attached and detached breast cancer cells (Figure S6A,B). Furthermore, BODIPY lipid uptake assays revealed that NNMT‐knockdown cells exhibited reduced lipid accumulation following lipid treatment, whereas NNMT‐overexpressing cells showed increased lipid deposits (Figure S6C), suggesting NNMT may also promote FAO by facilitating the fatty acid uptake. Taken together, these results support the notion that NNMT could enhance fatty acid oxidation in breast cancer cells, irrespective of attachment status.

### 2.5 NNMT Represses PP2A Activity to Induce FAO‐Mediated Anoikis Resistance

**FIGURE 5:**
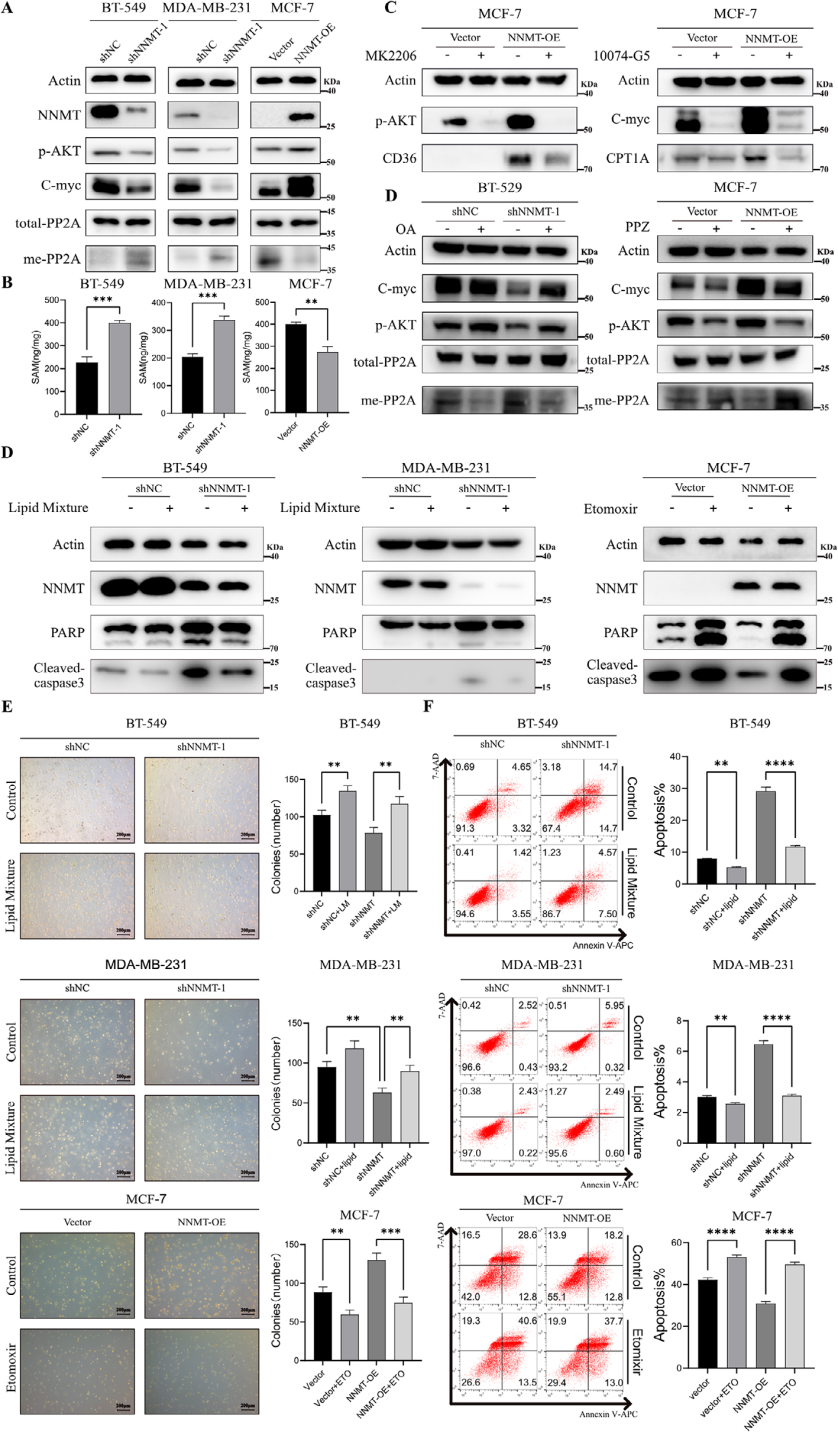
NNMT represses PP2A activity to induce FAO‐mediated anoikis resistance. (A) Representative WB results of methyl‐PP2A, PP2A, p‐AKT, C‐myc, and NNMT expression in BT‐549, MCF‐7, and MDA‐MB‐231 cell models. (B) Results of SAM quantification by LC‐MS/MS in three cell models by the LC‐MS/MS platform. Results showed that NNMT knockdown increases the SAM concentration and NNMT overexpression significantly reduces intracellular SAM pools in breast cancer cells. (C) Representative result of p‐AKT, C‐myc, CD36 and CPT1A proteins by western blotting in the MCF‐7 cell model with MK2206 (1 µm for 48 h) and 10074‐G5 (10 µm for 48 h) treatment. (C) Representative result of p‐AKT, C‐myc proteins by western blotting in MCF‐7 and BT‐549 cell model with PPZ (25 µm for 48 h) or OA (0.25 µm for 48 h) treatment. D‐F: Representative result of anoikis levels in BT‐549, MCF‐7, and MDA‐MB‐231 cell lines with etomoxir (100 µm for 48 h) or lipid mixture treatment (2% for 48 h) by flow cytometry, western blot, and soft agar assay. Data were presented as mean ± SEM. ^*^ *p* < 0.05, ^**^ *p* < 0.01, ^***^ *p* < 0.001, ^****^ *p* < 0.0001.

To investigate the impact of NNMT on the expression of CPT1A and CD36, we first examined whether NNMT affects their transcription factors, the PPARs pathway [34]. The results indicated that NNMT regulates CPT1A and CD36 in a PPARs‐independent manner (Figure S7A). So we hypothesized that the observed effects may be attributed to NNMT enzyme activity rather than the PPARs pathway. To investigate this, we constructed wild‐type NNMT as well as D197 and Y20 site mutations of NNMT, where the aspartic acid at position 197 and the tyrosine at position 20 in the NNMT protein sequence were mutated to alanine, respectively, which are implicated in affecting NNMT enzymatic activity [27, 35]. The results showed that CPT1A and CD36 expression in the NNMT mutant groups (D197 and Y20) was lower compared to the wild‐type NNMT group, indicating that the N‐methyltransferase activity of NNMT is required for its role in regulating CPT1A and CD36 (Figure S7B). However, stimulation with 1‐MNA, the metabolite of NNMT, did not induce a significant increase in CPT1A and CD36 (Figure S7C). Therefore, we suggested that the regulation of CPT1A and CD36 by NNMT may be achieved by the depletion of the methyl donor SAM. Then, we have incorporated LC‐MS/MS‐based quantification of intracellular SAM concentration, which demonstrates that NNMT overexpression significantly reduces intracellular SAM levels, whereas NNMT knockdown increases them (Figure 5B), providing direct experimental support for a SAM‐consumption mechanism.

PP2A is considered an important regulator of cancer development since it is a strongly conserved and a major protein phosphatase in mammalian cells [36], and methylation of the PP2A C‐terminal Leu309 residue in its catalytic center will enhance its activity. Recently, it was reported that PP2A was reported as the negative regulator of both AKT and c‐myc [37], while c‐myc is reported as a regulator of CPT1A [38], and P‐AKT is reported as a regulator of CD36 [39]. Therefore, we hypothesized that NNMT‐driven SAM depletion would lead to PP2A hypomethylation and functional inhibition. And we wondered if NNMT regulates FAO by the PP2A‐AKT/c‐myc axis. Firstly, we examined the methylation of PP2A expression when NNMT was knocked down or overexpressed. Results showed that the methylation of PP2A was negatively regulated by NNMT, with total PP2A unaffected (Figure 5A). Besides, the expression of c‐myc and P‐AKT decreased after NNMT knockdown, but increased after NNMT overexpression (Figure 5A). SiPP2A treatment reverses the NNMT knockdown‐induced attenuation of P‐AKT and c‐myc, indicating the role of PP2A in NNMT‐regulated c‐myc and P‐AKT axis (Figure S7E). Then, we treated NNMT overexpression MCF‐7 cells with c‐myc inhibitor 10074‐G5 and P‐AKT inhibitor MK2206. Results showed that the positive regulatory role of NNMT on CPT1A and CD36 expression was mainly achieved by upregulating c‐myc and P‐AKT (Figure 5C). In order to investigate whether PP2A methylation plays a critical role in the regulation of AKT and c‐myc, we treated BT‐549 and MCF‐7 cells, respectively, with the PP2A inhibitor okadaic acid (OA) and PP2A activator perphenazine (PPZ). The results confirmed our hypothesis that NNMT regulates FAO through PP2A‐mediated AKT and c‐myc activation (Figure 5D). To further validate these findings, cells were cultured in methionine‐deficient medium for 48 h. Liquid chromatography‐tandem mass spectrometry (LC‐MS/MS) analysis showed that this perturbation directly limits intracellular SAM concentration, which resulted in a marked upregulation of both CPT1A and CD36 protein levels (Figure S8), thereby effectively rescuing the downregulatory effect of NNMT knockdown.

Next, wondering whether NNMT induced anoikis resistance in breast cancer cells by promoting FAO, we treated NNMT‐overexpression MCF‐7 cells with FAO inhibitor etomoxir and NNMT‐knockdown BT‐549 and MDA‐MB‐231 cells with lipid mixture. As expected, the WB assay showed lipid mixture could attenuate anoikis caused by NNMT‐knockdown, and etomoxir increased anoikis in MCF‐7 NNMT‐overexpression cells, which were consistent with the results of the anoikis assay (Figure 5D). Meanwhile, the soft agar assay showed that etomoxir could significantly decrease the number of cell colonies in NNMT‐overexpression cells, whereas lipid mixture increased the number of colonies in NNMT‐knockdown cells (Figure 5E). The results of the anoikis assay also showed that etomoxir significantly impaired anoikis resistance in NNMT‐overexpression MCF‐7 cells (Figure 4F). Accordingly, lipid mixture could significantly inhibit anoikis of BT‐549 and MDA‐MB‐231 cells deficient in NNMT expression (Figure 4F). Taken together, these findings confirmed the hypothesis that NNMT could promote breast cancer cell anoikis resistance by regulating FAO.

### 2.6 NNMT‐Mediated FAO Induced Anoikis Resistance by Maintaining Redox Homeostasis

**FIGURE 6:**
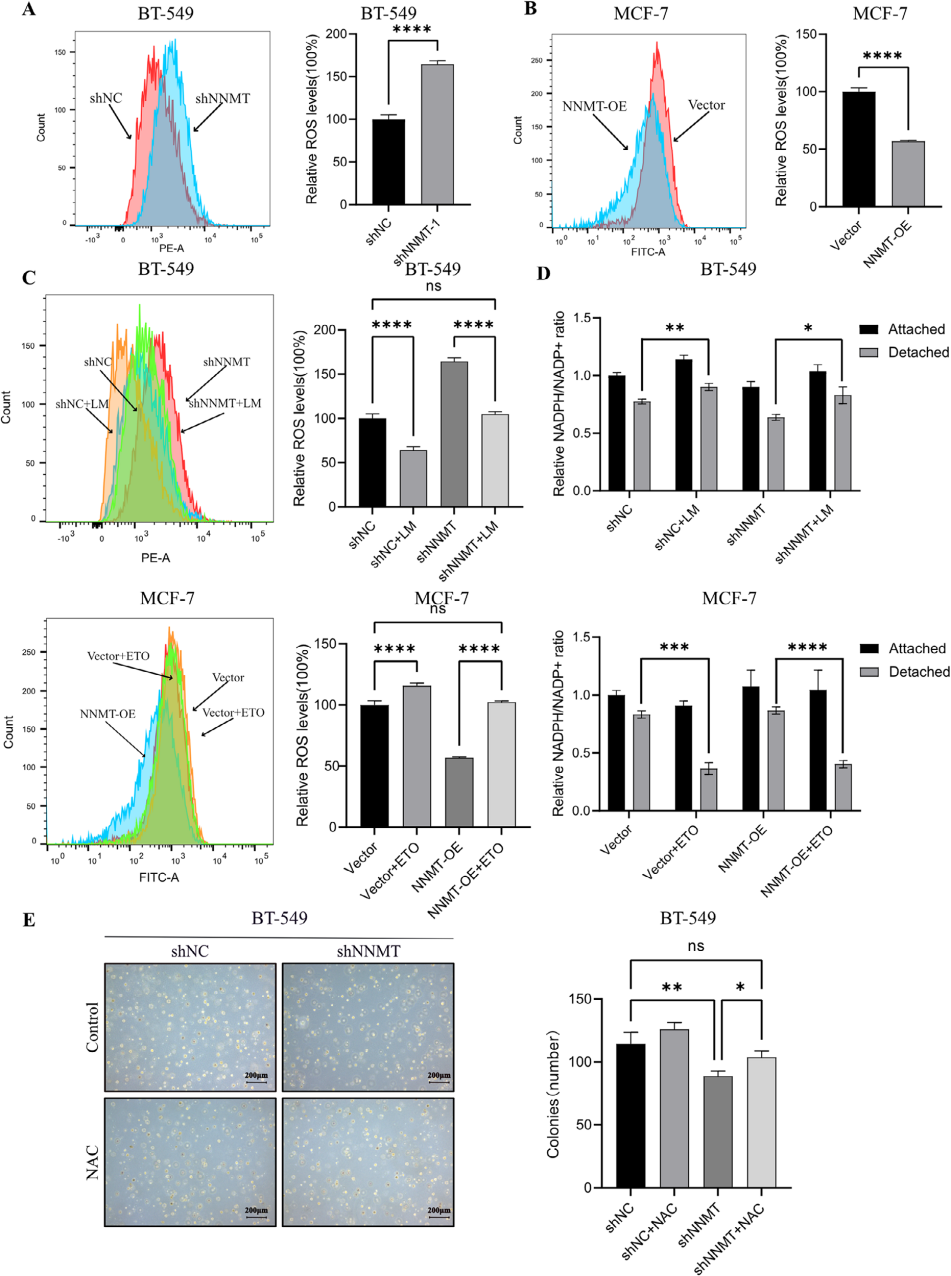
NNMT‐mediated FAO‐induced anoikis resistance by ROS clearance. (A) A graphical description of the relationship between NNMT, FAO, and ROS elimination. (B) Representative result of basic ROS levels in detached BT‐549 and MCF‐7 cell lines by DCF‐DA or DHE staining. ^****^ *p* < 0.0001. (C) Representative result of ROS levels in detached BT‐549 and MCF‐7 cell lines after etomoxir or lipid mixture treatment by DCF‐DA or DHE staining. ^**^ *p* < 0.01, ^***^ *p* < 0.001, ^****^ *p* < 0.0001. D: The NADPH/NADP+ ratio was determined in the attached and detached MCF‐7 and BT‐549 cell models. ^*^ *p* < 0.05, ^**^ *p* < 0.01, ^***^ *p* < 0.001, ^****^ *p* < 0.0001. (E) NAC, as a ROS scavenger, increased the clone number of the BT‐549 cell model in the soft agar assay. Data were presented as mean ± SEM. ^*^ *p* < 0.05.

Compared with attached cancer cells, detached cells exhibit a higher level of ROS, which triggers anoikis and hinders cancer metastasis [40]. Therefore, metastatic cancer cells always reduce oxidative stress to remain viable during detachment [41].FAO is a crucial source of cellular NADPH, which scavenges ROS indirectly by the glutathione system [42] and the thioredoxin system [43]. Thus, we measured ROS levels in detached breast cancer cells. As expected, ROS accumulation was much higher in detached BT‐549 NNMT‐knockdown cells than in the control group (Figure 6A), whereas detached NNMT‐overexpression MCF‐7 cells showed less ROS accumulation (Figure 6B). Also, ROS accumulation decreased in BT‐549 NNMT‐knockdown cells treated with lipid mixture, while etomoxir treatment significantly restrained the effect of NNMT on ROS clearance in NNMT‐overexpression MCF‐7 cells (Figure 6C). Furthermore, the NADPH/NADP^+^ ratio in BT‐549 NNMT‐knockdown cells did not persist after detachment (Figure 6D), which was consistent with the higher ROS accumulation observed in detached NNMT‐knockdown BT‐549 cells. In contrast, NNMT‐overexpressing MCF‐7 cells were more capable of maintaining redox homeostasis by stabilizing NADPH/NADP^+^ ratio, and this effect was suppressed by etomoxir (Figure 6D). Moreover, NAC (N‐acetyl‐L‐cysteine) treatment, one of the ROS scavengers, rescued the number of colonies of BT‐549 shNNMT cells in the soft agar assay (Figure 6E). These results demonstrated that ROS elimination via NNMT‐mediated FAO was critical to anoikis resistance in breast cancer cells and, further, that targeting this pathway may hold therapeutic potential.

### 2.7 Targeting NNMT‐FAO Axis Decreased Metastatic Formation In Vivo

**FIGURE 7:**
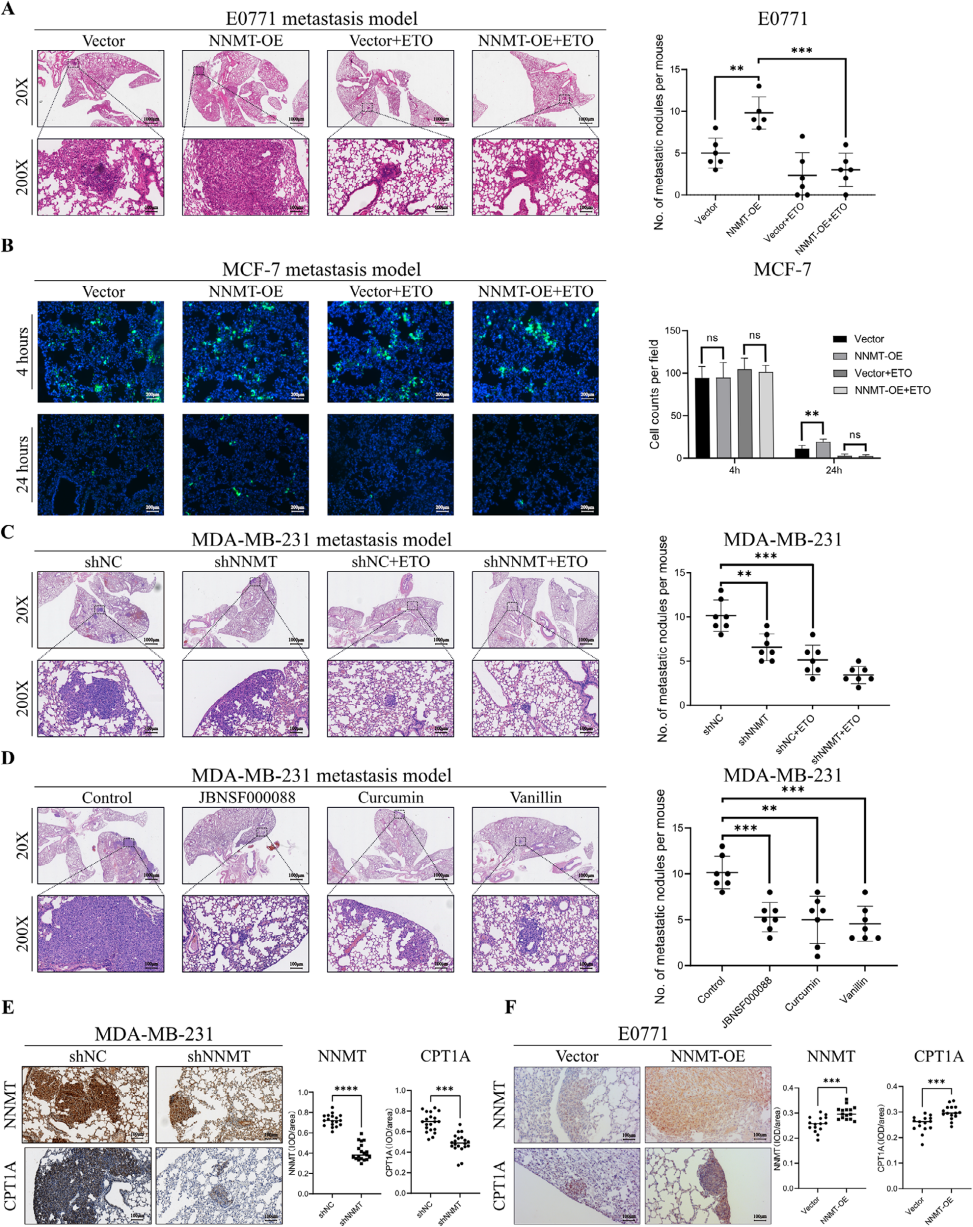
Targeting NNMT‐FAO axis decreased metastatic nodule formation in vivo. (A) The image of Lungs harvested after injecting vector and NNMT‐OE E0771 cells through the tail vein (*n* = 6 for each group) and HE staining for metastasis in lungs. Scale bar (20×), 1000 µm. Scale bar (200×), 100 µm. (B) Representative lung section images at 4 and 24 h after the Cell tracker Green labeled MCF‐7 cells injection (*n* = 5 for each group). Scale bar, 200 µm. (C) The image of Lungs harvested after injecting shNC and shNNMT MDA‐MB‐231 cells through the tail vein (*n* = 7 for each group) and HE staining for metastasis in lungs. Scale bar (20×), 1000 µm. Scale bar (200×), 100 µm. (D) The lung HE staining image of the mice metastasis model after NNMT inhibitor JBNSF000088, Vanillin, and Curcumin treatment. Scale bar (20×), 1000 µm. Scale bar (200×), 100 µm. (E,F) Representative result of CPT1A and NNMT expressions in lung metastasis in E0771 and MDA‐MB‐231 mice model by IHC. Scale bar, 100 µm.

To investigate the role of the NNMT‐FAO axis in breast cancer cell metastasis in vivo, we constructed lung metastasis models with breast cancer cell lines. In the E0771 lung metastasis model, NNMT overexpression increased the number of metastatic nodules, and treatment with etomoxir significantly suppressed the increase of metastatic lesions caused by NNMT‐overexpression (Figure 7A). To visualize the effects of NNMT on MCF‐7 cell anoikis resistance in vivo, we labeled MCF‐7 vector or NNMT overexpression cells with CellTracker Green and injected them into SCID mice via the tail vein. The results revealed that NNMT overexpression significantly increased the number of cancer cells in the lungs 24 h after injection, while etomoxir treatment suppressed the increase of survival cell number (Figure 7B). These findings demonstrated that NNMT‐mediated FAO played an essential role in the anoikis resistance of breast cancer, thereby promoting metastasis. The results of the MDA‐MB‐231 lung metastasis model showed that mice injected with NNMT‐knockdown cells exhibited fewer pulmonary metastatic nodules than the control group and etomoxir treatment also significantly inhibited pulmonary metastatic nodule formation in MDA‐MB‐231 lung metastasis model (Figure 7C). To determine whether pharmacological inhibition of NNMT could promote anoikis in breast cancer cells in vitro, we used JBNSF‐000088, a widely reported NNMT inhibitor, along with our previously reported inhibitors Curcumin [44] and Vanillin [23], all of which effectively induced anoikis in vivo (Figure S9). The results showed the promising therapeutic potential of inhibiting NNMT in metastatic breast cancer treatment (Figure 7D). To assess the effect of NNMT on FAO, we measured CPT1A and NNMT expression in the lung metastases of mice (Figure 7E,F). CD36 was too faint to be detected in MDA‐MB‐231 cells, which is consistent with the results in the cell model. Therefore, targeting the NNMT‐FAO axis could effectively stimulate anoikis and suppress breast cancer metastasis.

According to our findings, the upregulation of NNMT in detached cancer cells may be caused by the activation of the FAK‐STAT3 axis, and NNMT plays an essential role in the fatty acid metabolism of detached breast cancer cells to resist anoikis. Targeting the NNMT‐FAO axis may be able to provide new therapeutic targets for metastatic breast cancer treatment.

## 3 Conclusion

Circulating tumor cell are cancer cells shed from the primary tumor and able to survive in the peripheral blood, which are considered as potential indicators of tumor recurrence and can provide insights into the process of tumor metastasis [45]. CTCs encounter many risk factors in the vasculature including fluid shear stress, anoikis, and immunosurveillance. Therefore, CTCs must adapt their survival strategies to endure the hazardous vascular microenvironment [46]. Although CTCs originate from primary tumors, their gene expression profiles differ from those of primary tumor cells due to the different microenvironments they encounter [47].In this study, we found for the first time that NNMT was highly expressed in breast cancer CTCs, and the proportion of breast cancer patients with NNMT^+^ CTCs was 90%, much higher than our previous results in primary tumors (46%) [25], suggesting a possible association between NNMT and breast cancer CTCs formation. Since CTCs can be shed from primary tumors or from metastatic tumor sites, the elevated NNMT expression observed in CTCs may represent the phenotype of tumor cells originating from metastatic niches rather than de novo activation during CTC formation, which still needs further research. In this study, we found that NNMT promotes the aggregation of detached breast cancer cells. Western blot and qPCR analyses further revealed that NNMT upregulates the expression of key cell adhesion molecules, ICAM‐1 and CD44 (Figure S10). These findings suggest that NNMT may also play a role in the formation of CTC clusters. Besides, several studies have recently reported the immunosuppressive effects of NNMT and its metabolite 1‐MNA [48, 49], indicating that NNMT may be involved in the process of CTCs evasion of immune surveillance. Clarifying the biological role of NNMT in CTCs survival can help us gain insight into the mechanisms of tumor metastasis and may provide novel therapeutic options in invasive breast cancer treatment.

Anoikis is a specific form of programmed cell death triggered by cell detachment from the ECM, working as a critical protective mechanism in preventing adherent‐independent cell survival and attachment to an inappropriate ECM [41]. The activation of the FAK pathway is one of the most common mechanisms for achieving anoikis resistance in cancer cells [8]. Our study revealed that NNMT was upregulated by the FAK‐STAT3 axis in detached breast cancer cells, suggesting that NNMT may participate in the FAK‐mediated anoikis resistance. Consistently, our in vitro experiments showed that NNMT knockdown led to decreased survival of detached breast cancer cells, while NNMT overexpression resulted in enhanced survival and anoikis resistance of the detached breast cancer cells. Interestingly, our findings demonstrated that BT‐549 and MDA‐MB‐231 cells, which express high levels of NNMT, exhibit significantly lower levels of anoikis compared to MCF‐7 and HCC1937 cells, which express lower levels of NNMT. Since its discovery in 1984, NNMT has been reported to participate in cancer development and progression [50, 51]. Substantial evidence indicates that NNMT depletes the methyl donor S‐adenosylmethionine (SAM), thereby competitively inhibiting methylation processes of DNA, RNA, and proteins [24, 51]. PP2A, a well‐established methylation‐regulated serine/threonine phosphatase with critical roles in tumor suppression, has also been reported to be regulated by NNMT in this way [25]. The decrease in PP2A methylation has been shown to promote basal ERK pathway activity in cancer cells. This study aimed to investigate the function of NNMT in breast cancer CTCs, and we revealed that NNMT can upregulate CPT1A and CD36 by suppressing PP2A methylation. Moreover, our previous studies have shown that the catalytic product of NNMT, 1‐MNA, could attenuate ROS production by the ASK1‐p38 MAPK pathway [52]. In this study, our data show that the ROS scavenging effect of NNMT may be partially achieved through enhancing FAO, whereas 1‐MNA has no obvious effect on CPT1A or CD36 (Figure S7C). These findings may provide a novel perspective in understanding the role NNMT plays in cancer metastasis.

Metabolic reprogramming is required to meet the energy demands of cancer cells to support tumor metastasis. The metabolic switch from anabolism (glycolysis and fatty acid synthesis) to catabolism (FAO) is required to help cells evade anoikis [53]. FAO confers a critical survival advantage for cancer development by supplying NADH, NADPH, and ATP. Colorectal cancer uses FAO‐derived energy to resist anoikis while spreading to the liver [54], suggesting that FAO may play a critical role during ECM detachment. Metastatic HCC cells reprogrammed the anabolic/catabolic balance from aerobic glycolysis toward AMPK‐activated FAO to resist anoikis [55]. CPT1A, the FAO rate‐limiting enzyme, has been shown to facilitate esophageal squamous cell carcinoma cells in resisting anoikis by maintaining redox homeostasis [56]. Accordingly, our study demonstrates that NNMT may mediate the metabolic reprogramming toward FAO in breast cancer. However, the role of NNMT in fatty acid metabolism is still controversial. Studies have shown that NNMT can regulate hepatic nutrient metabolism by stabilizing Sirt1 and that liver NNMT expression inversely correlates with total cholesterol, LDL cholesterol, and TG levels in serum, as well as other parameters in both human and mice [57]. Furthermore, nicotinamide, the NNMT substrate, may cause loss of body weight in rats by reducing food intake and food efficiency decrease at pharmacological doses [58]. Conversely, 1‐MNA, the NNMT product, can improve the metabolic profile of mice fed with a high‐fat diet [59]. Other researchers hold conflicting views. For instance, Kraus reported that NNMT knockdown in white adipose tissue and liver protected against diet‐induced obesity by increasing cellular energy expenditure [60], and Xu demonstrated that NNMT promoted lipid accumulation in 3T3‐L1 adipocytes [61]. Therefore, further experiments are needed to clarify the specific function of NNMT. In this study, we found that NNMT could upregulate CPT1A, the FAO rate‐limiting enzyme, and CD36, one of the lipid uptake receptors. Therefore, NNMT may contribute to both lipid catabolism and lipid accumulation. The balance of cellular fatty acid oxidation and uptake mediated by NNMT in various tissue types results in distinct metabolic behaviors toward fatty acids, which may explain the controversial effects of NNMT in lipid metabolism. In breast cancer cells, BODIPY staining confirmed that NNMT ultimately promotes fatty acid utilization (Figure S11) and NNMT induced fatty acid uptake may also have a contributing effect on FAO. Tumors are well‐known as heterogeneous entities consisting of cancer cells with varying metabolic states [62]. It is also well recognized that cancer cells can optimize the exploitation of limited energy resources, particularly when cells undergo ECM detachment [40].

Cancer cells rely on the reprogramming of cellular metabolism to facilitate malignant growth [63]. Therefore, targeting cancer metabolism is an emerging crucial issue in clinical cancer therapy. Recently, FAO has received increasing attention in the field of tumor therapy. Several studies have shown that FAO is indispensable for tumor progression and can be a novel metabolic target for cancer therapy [64, 65]. In this study, we probed that NNMT could induce breast cancer anoikis resistance by enhancing the FAO rate. Our data also revealed that FAO inhibitor etomoxir could reverse anoikis resistance induced by NNMT in the breast cancer metastasis model. Although etomoxir has been reported to induce myocardial hypertrophy and hepatocellular injury [66], obvious weight reduction is not observed (Figure S12A). Additionally, we investigated the impact of the NNMT antagonists JBNSF000088, Curcumin, and Vanillin on the breast cancer metastasis model. Results showed that all three inhibitors significantly suppressed breast cancer metastasis in vivo. Overall, our results demonstrate that inhibiting the NNMT‐FAO axis represents a promising strategy for metastatic breast cancer treatment.

Collectively, we identified that high expression of NNMT in breast cancer CTCs contributes to anoikis resistance by enhancing FAO. Mechanically, NNMT inhibits PP2A methylation by depleting methyl donors SAM, thereby regulating CPT1A and CD36 expression. Furthermore, in detached breast cancer cells, the FAK‐STAT3 axis could activate NNMT transcription, resulting in a high positive proportion of NNMT in CTCs. These findings highlight the significant role of NNMT in the survival of breast cancer CTCs. Consequently, further research and drug development efforts targeting NNMT may hold crucial implications for the diagnosis and treatment of invasive breast cancer.

## 4 Experimental Section

### 4.1 Cell Lines, Reagents and Antibodies

Human breast cancer cell lines BT‐549(RRID: CVCL_1092), MDA‐MB‐231(RRID: CVCL_0062), HCC1937(RRID: CVCL_0290), MCF‐7(RRID: CVCL_0031), and E0771(RRID: CVCL_GR23) were purchased from the American Type Culture Collection (ATCC, USA). MDA‐MB‐231, MCF‐7, and E0771 were cultured in DMEM medium (Gibco, USA). HCC1937 cells were grown in RPMI1640 medium (Gibco), and BT‐549 cells were cultured in RPMI1640 medium containing 0.023 U/mL insulin. All mediums were supplemented with 10% FBS (Gibco), 100 U/mL of penicillin, and streptomycin (Sigma–Aldrich). Cells were maintained in a humidified incubator supplemented with 5% CO_2_ at 37°C. Prior to the experiments, these cell lines were confirmed to be free of mycoplasma contamination.

Lipid mixtures were obtained from Sigma–Aldrich(#L2088). BODIPY FL C16 was purchased from Thermo Fisher Scientific(#D3821). DCF‐DA was purchased from Thermo Fisher Scientific(#D2935). DHE was purchased from Applygen (#C1300‐2). PF‐562271(#S2890), MK2206(#S1078), 10074‐G5(#S8426), and C188‐9(S8605) were purchased from Selleckchem. PPZ(HY‐A0077A) and OA (#HY‐N6785) were purchased from MCE. The antibodies used for Western blot analysis were as follows: anti‐β‐actin (#4970), anti‐P‐STAT3(Tyr705) (#9145), anti‐STAT3(#9139), anti‐FAK(#3285), anti‐P‐FAK (Tyr397) (#8556), anti‐CPT1A (#12252), anti‐CD36 (#14347), anti‐P‐AKT(Ser473) (#4060), anti‐AKT(#9272), anti‐PARP(#9532), anti‐C‐myc(#18583), anti‐PP2A(#2038), anti‐PPARγ(#2443), anti‐ACC(#3676), anti‐P‐ACC(#11818), anti‐LXRβ(#13519) (Cell Signaling Technology), anti‐PPARα(ab126285), anti‐FGF21(ab171941) (Abcam), anti‐methylation‐PP2A(sc‐81603) (Santa Cruz Biotechnology), anti‐LXRα(14315‐1‐AP) (Proteintech), and anti‐NNMT(1E7) obtained as previously described [26].

### 4.2 Lentiviral Infection and siRNA Transfection

For lentiviral infection, cells were seeded overnight in six‐well plates (2 × 10^5^ cells/well) and then co‐cultured with lentivirus carrying shRNA targeting human NNMT (GeneChem Co., Ltd., Shanghai), as previously described [25]. The shRNA sequences targeting NNMT are provided in Table S7. Following infection or transfection, cells were maintained in fresh complete medium for further experiments.

### 4.3 Patients’ Characteristics and Blood Sample Harvest

This study was approved by the Human Research Ethics Committee of Sir Run Run Shaw Hospital (Permit Number: 20210210–222). 2 mL blood samples from 45 newly diagnosed breast cancer patients were collected for CTCs isolation and quantification. The diagnoses of 45 patients were confirmed by pathologic results of breast biopsy from August 4, 2023, to January 31, 2024, at Sir Run Run Shaw Hospital (Hangzhou, China). As shown in Tables S1 and S2, the clinical characteristics of these patients were extracted from their medical records, including age, TNM stage (tumor diameter, lymph node metastasis and distant metastasis), ER, Her‐2, and PR according to the guideline for breast cancer of the Chinese society of clinical oncology (Version 2020).

### 4.4 CTCs Isolation and Quantification

As previously reported [67], for human samples, CTC detection was performed using the CytoSorter system (Hangzhou Watson Biotech, Hangzhou, China), a microfluidic‐based immuno‐capture platform. Briefly, blood samples were diluted 1:1 with PBS, and transferred into Leucosep tubes containing 2 mL of Histopaque‐1077 density gradient media. After centrifugation, the mononuclear cell layer was resuspended in 190 µL of the same washing medium and deposited on CytoChipNano that has been pre‐treated with Pan‐CK capture antibody at a concentration of 3 × 106 cells/slide. The enrichment procedure was controlled by CytoSorter software. Once the CTC enrichment was finished, the CytoChipNano was removed from the CytoSorter device and proceeded to the manual immunofluorescence staining with Pan‐CK, CD45, NNMT, and DAPI. For mouse samples, RBC lysis was performed with ammonium chloride solution. After centrifugation, nucleated cells were seeded onto glass slides (up to 12 slides at 3 × 10^6^ cells per slide) and subjected to immunofluorescence staining for Pan‐CK, CD45, NNMT, and DAPI. An automated fluorescence microscope (Nikon) was used to verify the localization and staining of human and mouse CTCs. CTCs were defined as Pan‐CK^+^, CD45^−^ and DAPI^+^. Pan‐CK^−^, CD45^+^ and DAPI^+^ cells indicate white blood cells. This study was approved by the Human Research Ethics Committee of Sir Run Run Shaw Hospital (Permit Number: 2023‐0427).

### 4.5 Public Data Set Analysis

To explore whether NNMT is expressed in the nesting cells of breast cancer, we collected sequencing data from three sources of breast cancer, namely, in situ tissue, peripheral blood, Circulating tumor cells, and metastatic lesions such as metastatic breast cancer. RNA‐seq sequencing data of in situ breast cancer and metastatic breast cancer are from GSE209998; CTCs and metastatic breast cancer sequencing data are from GSE113890. After the conversion of raw counts to FPKM data of each cohort, we used the “sva” package in R to adjust gene expression in all samples. We then used PCA to visualize the status of batching, finally getting the gene expression of three positions. Table S3 summarizes the corrected data from 2 cohorts.

Through the combined cohort (GSE209998 and GSE113890), we used the limma package for differential gene analysis on the sequencing results of CTCs versus in situ tissue source samples, using the following standard logFC>0 and P<0.05. The significant differentially expressed gene (DEG) of its CTCs group was analyzed by enrichGO for pathway function enrichment, and the criterion of pathway difference was q‐value<0.05 and p‐adjust<0.05.

### 4.6 DEG Analysis of Attached and Detached Breast Cancer Cells

Total RNA was extracted from the attached MDA‐MB‐231 and detached MDA‐MB‐231 cell model. After quality control of RNA amount, purity, and integrity, a cDNA library was generated from total RNA. The library was sequenced on an Illumina Novaseq 6000. Differentially expressed genes were defined as fold change >2 or fold change <0.5 and *p* < 0.05. All services were provided by LC Biotech Corporation (Hangzhou, China). Table S4 lists the results of the differentially expressed genes in detached MDA‐MB‐231 cells compared to the attached group.

### 4.7 Soft Agar Assay

Before the experiment, solidified culture medium containing 0.7% agarose was poured into a six‐well plate. 4–6 × 10^4^ breast cancer cells and reagent (lipid mixture or etomoxir) were mixed with culture medium containing 0.35% agarose before being added to the solidified culture medium. After solidification, 1 mL culture medium with lipid mixture or etomoxir was added to the top layer. The culture medium containing the reagent was changed every 2–3 days. After 2–3 weeks, images were taken with a Carl Zeiss Microscope.

### 4.8 Anoikis Assay

The proportion of detached breast cancer cells undergoing anoikis was determined using a 7‐AAD/PI kit (Multi Science, 70‐AP105‐100). Cells were cultured on a 1% agar‐treated plate as described [68] for 48 h. First, cells were collected and washed twice with PBS. Then cells were stained away from light for 30 min at room temperature, before analysis by flow cytometry (FACSCalibur flow cytometer; BD Biosciences).

### 4.9 FAO Pathway Correlation Analysis

Three breast cancer cohorts, GSE4922, GSE9893, and GSE24450, were normalized and combined to detect the correlation between NNMT and FAO pathways. The functional gene of the FAO pathway was derived from the MSigDB database. Its full name was FATTY_ACID_OXIDATION, which contained 18 genes, including ACADM, ACADS, ACADVL, ADIPOR1, ADIPOR2, ALOX12, BDH2, CPT1A, CPT1B, ECH1, ECHS1, HACL1, HADHB, HAO1, HAO2, PPARA, PPARD, and PPARGC1A. The single sample enrichment analysis (ssGSEA method) was used to analyze all chips or sequencing data. Table S6 summarizes the significantly enriched pathways with adjusted P‐values (P‐adjust < 0.001).

### 4.10 Seahorse FAO Analysis

FAO rate was measured by the Seahorse XF96e Extracellular Flux Analyzer (Agilent Technologies). Breast cancer cells were incubated on agar plates or normal plates for 48 h before being harvested. Briefly, breast cancer cells were first cultured in XF cell culture microplates with the typical medium. Before starting, cells were cultured in the substrate‐limited medium for 16 h. At 45 min before the assay, cells were washed twice and then cultured with FAO assay medium. Just before starting the assay, 30 µL XF palmitate‐BSA, as FAO substrate, or BSA was added. Finally, the oxygen consumption rate (OCR) was detected with Mito stress test protocols on Seahorse XF96e Extracellular Flux Analyzer. The FAO rate was normalized to protein levels in each well.

### 4.11 BODIPY Staining Assay

To measure lipid deposit, breast cancer cells were first stained with PBS containing 0.1 µg/mL BODIPY FL C16 for 20 min at 37°C. After incubation, cells were washed with PBS 3 times, followed by paraformaldehyde fixation. Then cells were stained with DAPI. Images were taken with a confocal microscope. For the lipid uptake assay, breast cancer cells were first cultured in serum‐free medium for 18 h. Then, cells were treated with 2% lipid mixture for 4 h. After incubation, cells were washed with PBS for 3 times, and BODIPY staining was performed as described above.

### 4.12 WB and QPCR

Total protein was extracted with RIPA buffer (Beyotime Biotechnology), and the concentration of protein was measured with the BCA kit (Multi Sciences). Then Western blot analysis was performed as described [25].

Total RNA was isolated with TRIzol reagent (Invitrogen). Then, RNA was reverse transcribed into cDNA using the HiFiScript cDNA Synthesis Kit (CWBio). Target mRNA levels were measured with the NovoStart SYBR qPCR SuperMix Plus Kit (Novoprotein). Relative expression levels were calculated using the 2^−ΔΔCt^ method. Data were normalized to β‐actin levels. The primer sequences used were listed in Table S8.

### 4.13 ChIP Assay

Samples for ChIP experiments were prepared using SimpleChIP Plus Enzymatic chromatin IP kit (#9005, Cell Signaling Technology) according to the manufacturer's protocol. ChIP experiments were performed with anti‐P‐STAT3 antibody (#9145, Cell Signaling Technology) and anti‐IgG. PCR and qPCR primers for the NNMT promoter are described, respectively in Table S7.

### 4.14 Dual‐Luciferase Reporter Assay

Dual‐luciferase reporter assays were performed using the Dual‐Luciferase Reporter Assay System (#E1960, Promega) following the manufacturer's instructions. All plasmids used in this assay, including the firefly luciferase reporter vectors (e.g., pGL4‐basic containing the wild‐type or truncated NNMT promoter), the STAT3 expression plasmid, and the Renilla luciferase internal control plasmid (pRL‐TK), were custom‐synthesized by GeneChem Co., Ltd. (Shanghai, China). Briefly, cells were co‐transfected with a firefly luciferase reporter plasmid (e.g., pGL4‐NNMT promoter‐WT or pGL4‐NNMT‐promoter‐Mut), a STAT3 expression plasmid or control vector, and a Renilla luciferase internal control plasmid (pRL‐TK). After 48 h, cells were lysed, and firefly and Renilla luciferase activities were measured sequentially using a microplate luminometer. Firefly luciferase activity was normalized to the Renilla luciferase activity for each sample to control for transfection efficiency.

### 4.15 Quantification of SAM by LC‐MS/MS

The intracellular levels of SAM were quantified by liquid chromatography‐tandem mass spectrometry (LC‐MS/MS) using an AB SCIEX Triple Quad 4500MD system. For sample preparation, 50 µL of cell lysate or a standard solution was mixed with 250 µL of 1% zinc sulfate solution containing the stable isotope‐labeled internal standard NMNA‑d4. After vortexing at 400 rpm for 30 min, samples were centrifuged at 14,000 rpm for 20 min, and the supernatant was transferred to glass vials for analysis. Chromatographic separation was achieved on an Eclipse XDB‑C18 column (4.6 × 150 mm, 5 µm; Agilent) coupled to a Jasper (SCIEX) LC system. The isocratic mobile phase, consisting of 0.1% formic acid in methanol (v/v), was filtered through a 0.22 µm membrane, degassed ultrasonically for 15 min, and delivered at a flow rate of 1 mL/min into the electrospray ionization (ESI) source. The retention times for SAM and NMNA‑d4 were 1.20 min and 1.65 min, respectively. Quantification was based on a standard curve, and final concentrations were normalized to the total protein content of the cell samples.

### 4.16 H&E Staining and Immunohistochemistry

First, tissue samples were fixed in formalin and embedded with paraffin. The paraffin‐embedded tissue samples were cut into 4µm‐thick sections and then baked at 65°C for 2 h. After deparaffinization and hydration, sections were treated with 0.01 m citrate buffer pH 6.0, and microwave heat induction. Then, the sections were treated with 1% H_2_O_2_ for 5 min. After washing with PBS, BSA was used to block non‐specific binding for 10 min. The sections were incubated with anti‐CPT1A (Abcam, ab128568, dilution 1:200) and anti‐NNMT (1E7, 1:200) in a moist chamber and then with a biotinylated secondary antibody for 30 min. Subsequently, the sections were visualized by freshly prepared diaminobenzidine (DAB) and counterstained with hematoxylin. Finally, the images were captured by the digital slide scanning system.

### 4.17 Measurement of Cellular ROS and NADPH/NADP^+^ Ratios

Briefly, breast cancer cells were collected and trypsinized as a single‐cell suspension. Then the cells were stained with 1 µm DCFH‐DA or DHE to avoid interference from the green fluorescence of the lentivirus. Subsequently, cells were incubated at 37°C for 30 min and washed twice with culture medium before detection via flow cytometry.

Total cellular NADPH/NADP+ ratios were determined by Amplite Colorimetric NADP/NADPH Ratio Assay Kit (AAT Bio, 15274). The experiments were performed according to the manufacturer's protocol.

### 4.18 Breast Cancer Mouse Models

Female SCID mice or C57/BL6 mice (6–8 weeks old) were obtained from the Model Animal Research Center of Nanjing University and housed under pathogen‐free conditions on a 12/12 h light/dark cycle with free access to food and water. The animal experiment was approved by the Institutional Animal Care and Use Committee of Sir Run Run Shaw Hospital. All procedures were performed in accordance with the ethical guidelines of the Declaration of Helsinki. For the breast cancer orthotopic injection model, 5 × 10^6^ MDA‐MB‐231 shNC or shNNMT cells suspended in 100 µL PBS mixed with matrix gel were injected into the fourth mammary fat pad of female SCID mice. 8 weeks later, the mice were sacrificed, and blood samples were harvested for CTCs detection. For the MDA‐MB‐231 metastasis model, 1.5 × 10^6^ MDA‐MB‐231 cells suspended in 100 µL PBS were injected into SCID mice through the tail vein. Vehicle or 40 mg/kg etomoxir was injected intraperitoneally every 2 days, and mice were weighed every week before sacrificed. 8 weeks later, the respective metastatic organs were harvested, and metastasis was confirmed by H&E staining. For the E0771 metastasis model, 5 × 10^5^ E0771 vector or NNMT‐overexpression cells suspended in 100 µL PBS were injected into C57/BL6 mice through the tail vein. vehicle or 40 mg/kg etomoxir was injected intraperitoneally every 2 days. The lungs were harvested for H&E staining and IHC after 2 weeks. For the MCF‐7 metastasis assay in vivo, the MCF‐7 vector and NNMT‐OE cells were labeled with CellTracker Green and injected into SCID mice via the tail vein. Before injection, MCF‐7 cells were treated with etomoxir or DMSO. Lung tissues were collected at 4 and 24 h after injection and then processed into frozen sections, which were stained with DAPI for fluorescence microscopy.

### 4.19 Statistical Analysis

Statistical analysis was performed using SPSS 22.0. Statistical significance between groups was expressed as mean ± standard deviation (SD) and calculated by a two‐tailed Student's test. Significance values are ^*^ *p* < 0.05, ^**^ *p* < 0.01, ^***^ *p* < 0.001, and ^****^ *p* < 0.0001. The difference analysis in NNMT RNA expression among multiple groups was assessed by one‐way ANOVA followed by the Bonferroni method using R software. In the sequencing cohorts, Spearman's rank correlation coefficient was used to correlate NNMT and FAO with a statistical difference of *P* < 0.05.

## Author Contributions

T.Q.C. was responsible for validation, formal analysis, writing the original draft and review & editing. M.Y.L. was responsible for investigation, writing the original draft and review & editing. G.Y.Z. was responsible for data curation, investigation and software. Z.L., L.L.P. and F.Q. were responsible for the investigation. F.S.N. was responsible for investigation and data curation. Z.R. was responsible for the investigation, data curation. Z.J. (Zeng Jin) was responsible for investigation and data curation. L.G.L. and Y.J. were responsible for methodology. X.X.Y. was responsible for the investigation and conceptualization. W.Y.Z. was responsible for validation, review, and editing. Z.J. (Zhang Jun) was responsible for conceptualization, supervision, funding acquisition, and review & editing.

## Funding

This work was supported by grants from the National Natural Science Foundation of China (82172362, 81271914, 82302612 and 81800023), China Scholarship Council (201906325012), Key Research and Development Program of Zhejiang Province (2019C03021 and 2022C03037), Natural Science Foundation of Zhejiang Province (LY24H200001 and LY22H200002), Health Bureau Foundation of Zhejiang Province (2024KY1104), The Medical Science and Technology Project of Zhejiang Provincial Health Commission (2022KY1186), Key Laboratory of Precision Medicine in Diagnosis and Monitoring Research of Zhejiang Province (2022E10018), and Medical and Health Science and Technology Project of Zhejiang Province (2023RC184).

## Conflicts of Interest

The authors declare no conflicts of interest.

## Supporting information




**Supporting File 1**: advs74779‐sup‐0001‐FiguerS1.docx.

**Supporting File 2**: advs74779‐sup‐0002‐Tables1.docx.

**Supporting File 3**: advs74779‐sup‐0003‐Tables2.xlsx.

## Data Availability

All data in this study are true and reliable. The data that support the findings of this study are available from the corresponding author upon reasonable request. The remaining figure data are uploaded as Supplementary information.
