# Supplementary material for: Detachment‐Induced FAK‐STAT3‐NNMT Inhibits CTCs Anoikis to Promote Breast Cancer Metastasis by Enhancing Fatty Acid Oxidation
Source: Adv Sci (Weinh). 2026 Mar 12;13(29):e22837. doi: 10.1002/advs.202522837 (PMC13205902; doi:10.1002/advs.202522837)
Supplement: Supplementary file 1 — Supporting File 1: advs74779‐sup‐0001‐FiguerS1.docx. [file ADVS-13-e22837-s003.docx]

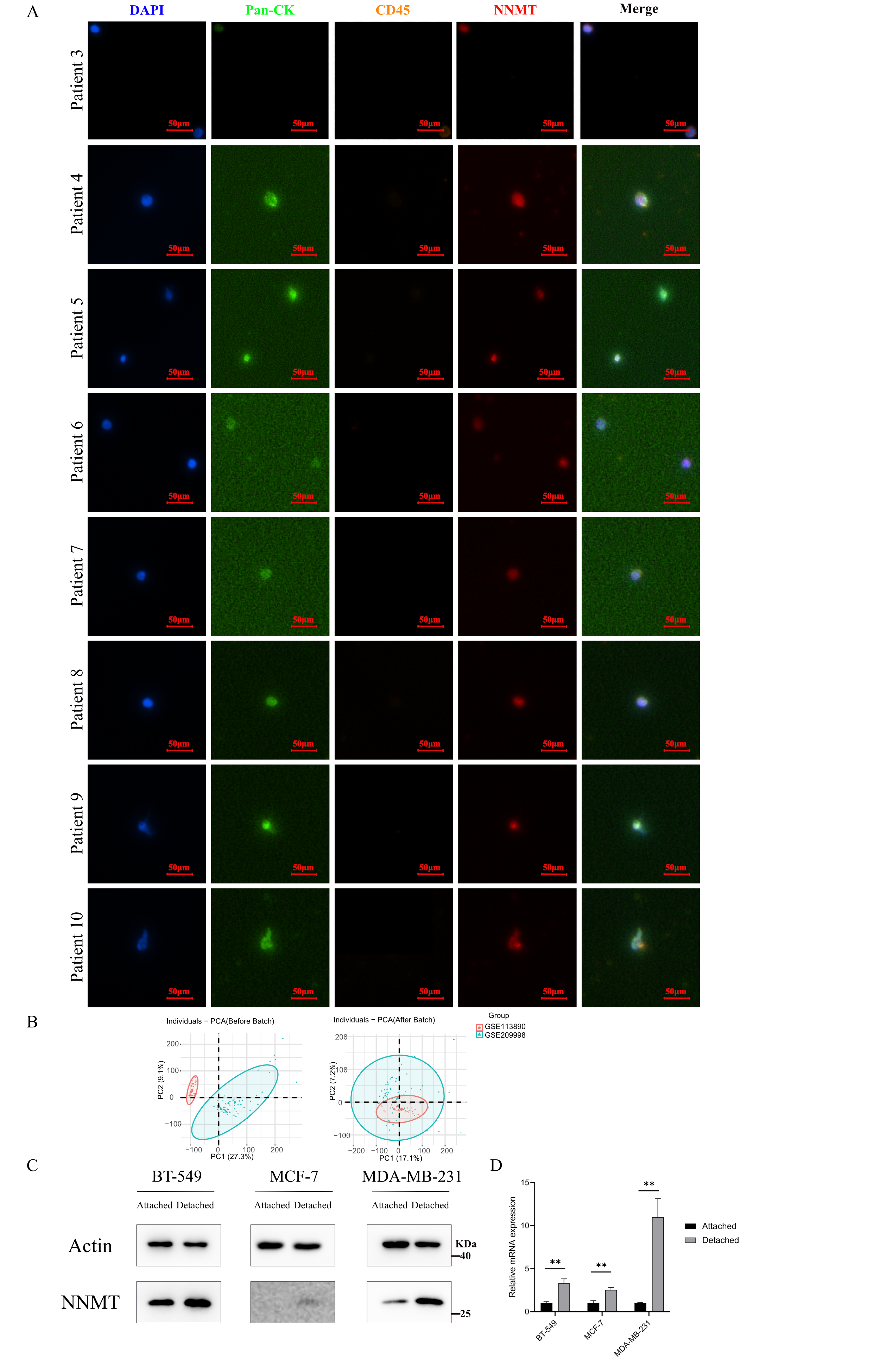


FigS1 A: Representative result of NNMT expression in breast cancer CTCs from the remaining 8 patients. Scale bars, 50μm. B: Representative plots of GSE209998 and GSE113890 datasets, batch-corrected using the SVA package, with PCA visualization confirming reduced technical variation. C-D: Representative result of NNMT expression by WB and QPCR in the detached BT-549, MCF-7, and MDA-MB-231 cells. Data were presented as mean ± SEM. **P < 0.01.


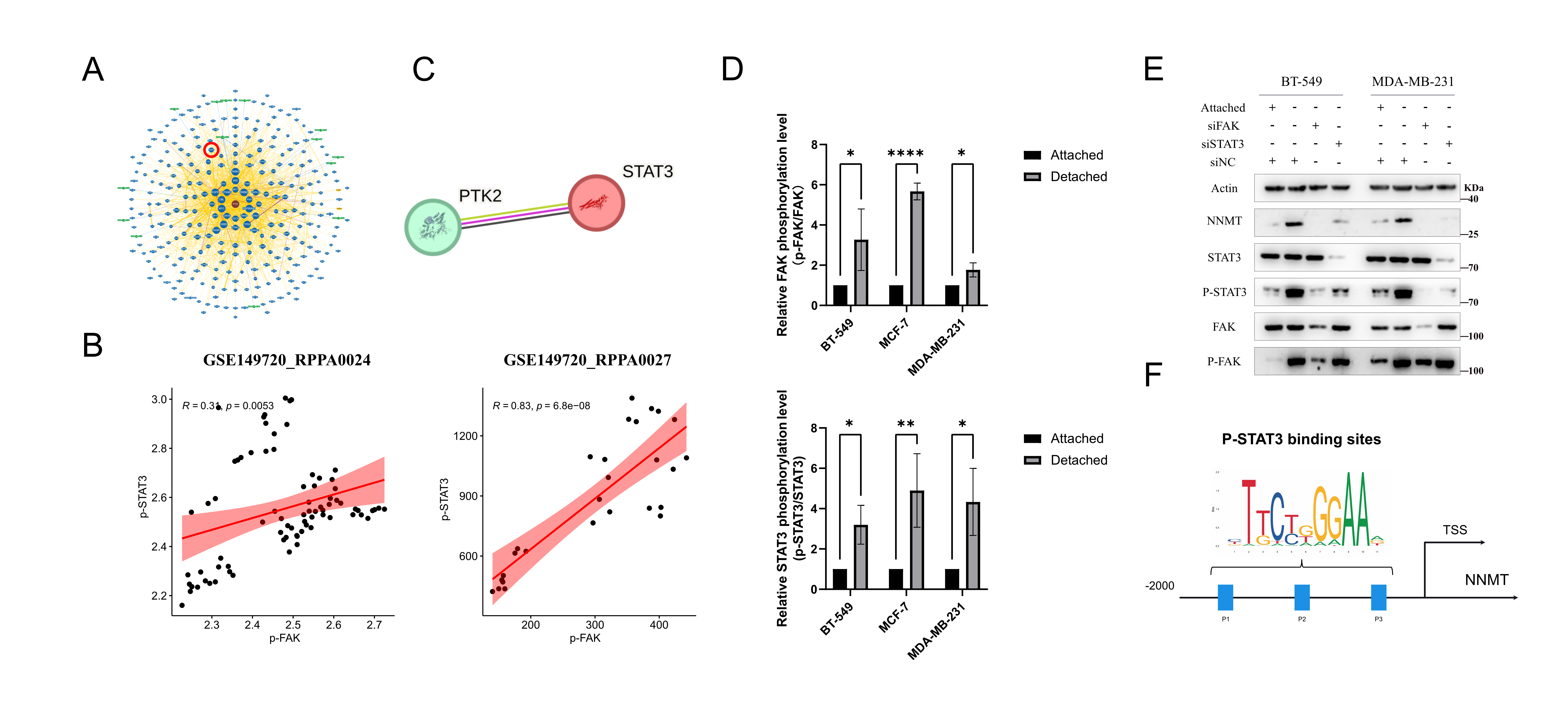


FigS2: A: Representative image of FAK interaction network according to the BioGRID 4.4 database analysis. B: Relationship between the expression of P-FAK and P-STAT3 in breast cancer patients from 2 batches of GSE149720. C: Representative image of the interaction of FAK and STAT3 according to the STRING database. D: Grayscale analysis of the P-FAK/FAK and P-STAT3/STAT3 bands from attached and detached breast cancer cells showed an increase in the phosphorylation levels of both FAK and STAT3. E: The representative WB results of FAK/STAT3 suppression by siRNA in BT-549 and MDA-MB-231 cultured under ultra-low attachment conditions. F: Representative image of the P-STAT3 binding sites in NNMT promoters based on JASPAR database analysis.


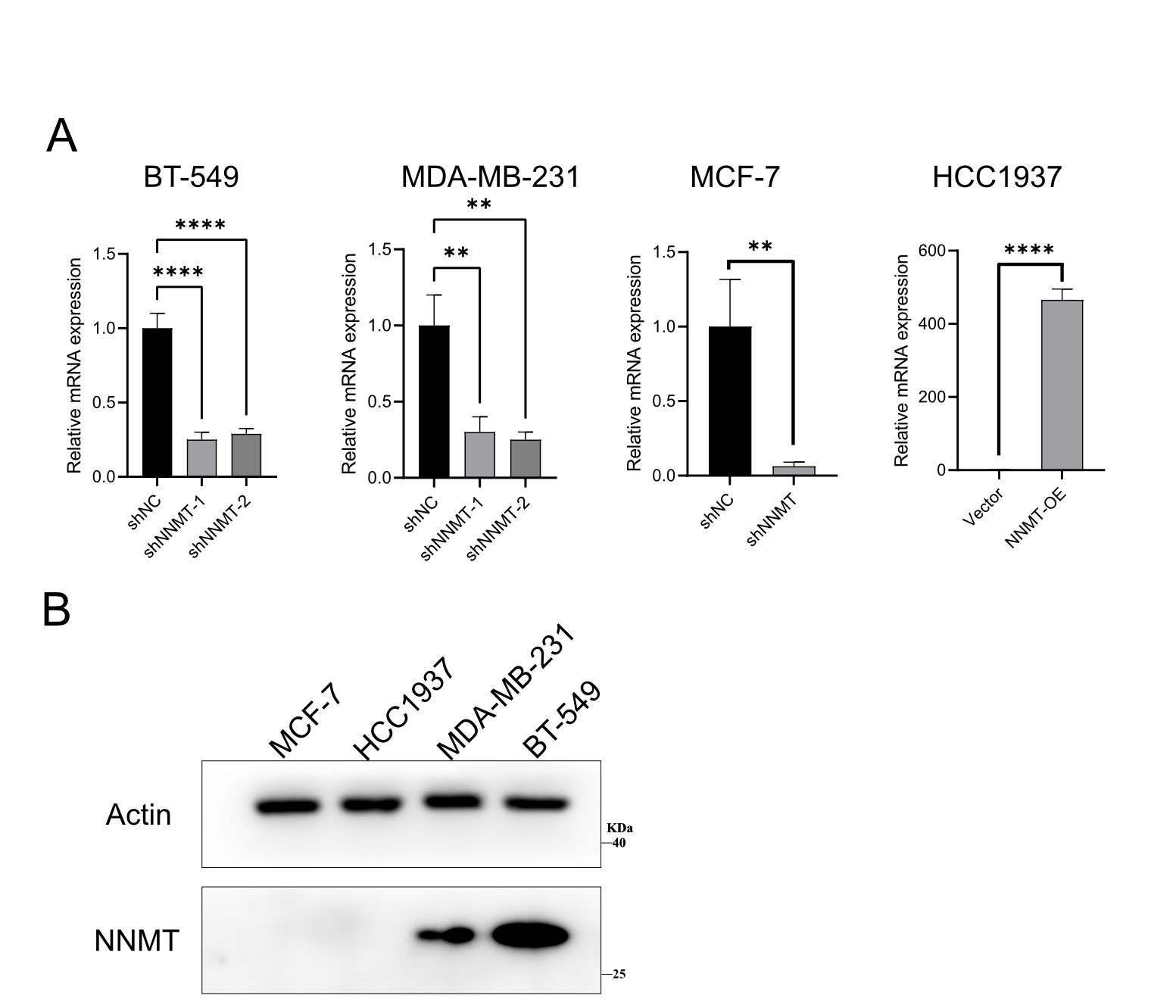


FigS3: A: The presentative results of NNMT protein levels by QPCR in four cell models used in anoikis assay. B: The presentative results of NNMT protein levels by Western blotting in four breast cancer cell lines used in this study.


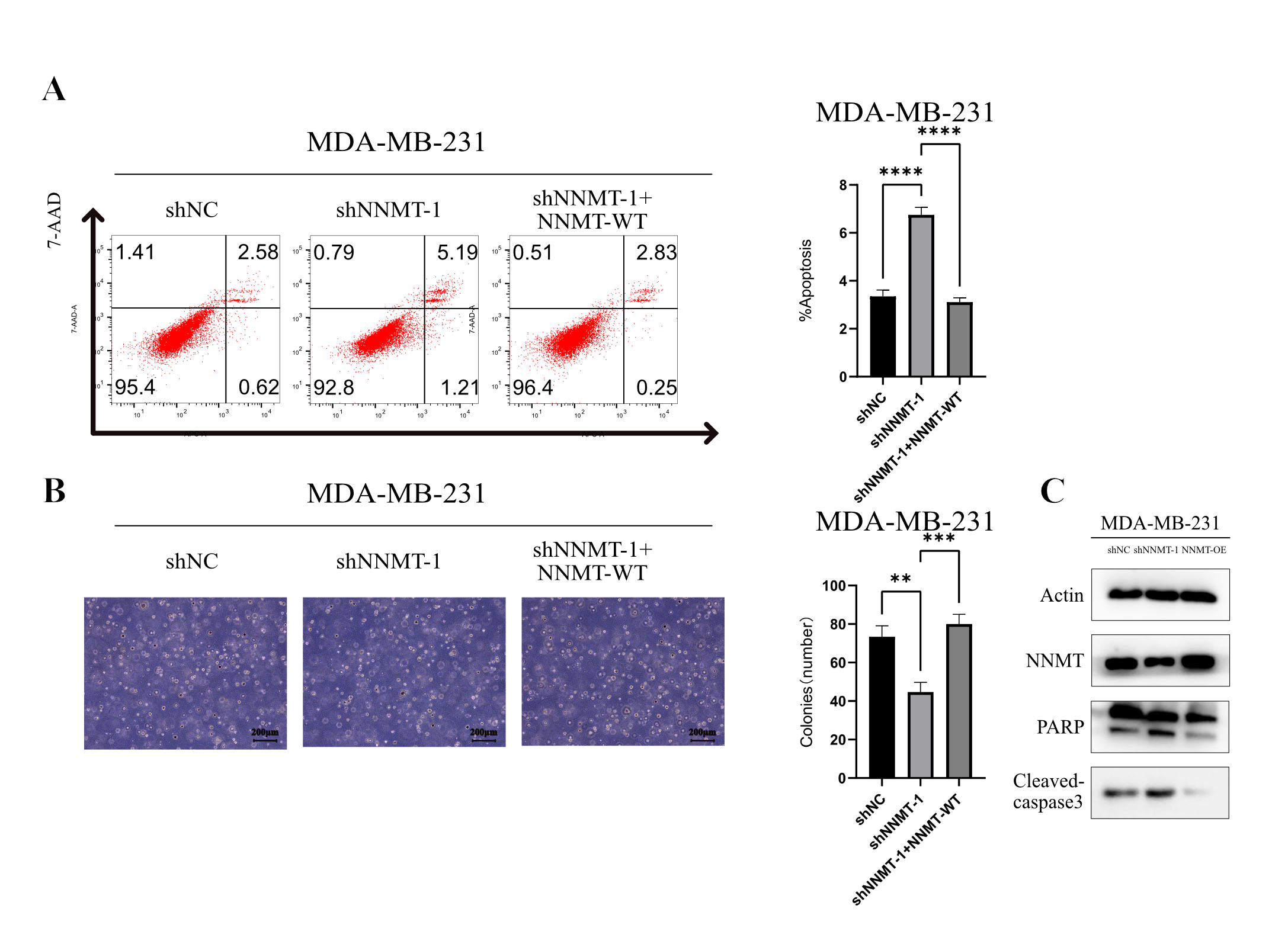


FigS4: Results of Anoikis analysis(A), soft agar colony formation assay(B),WB(C) in MDA-MB-231 cells with NNMT knockdown followed by NNMT re-expression. Re-introduction of NNMT significantly inhibited anoikis, confirming the specific role of NNMT in promoting cell survival under detachment conditions.


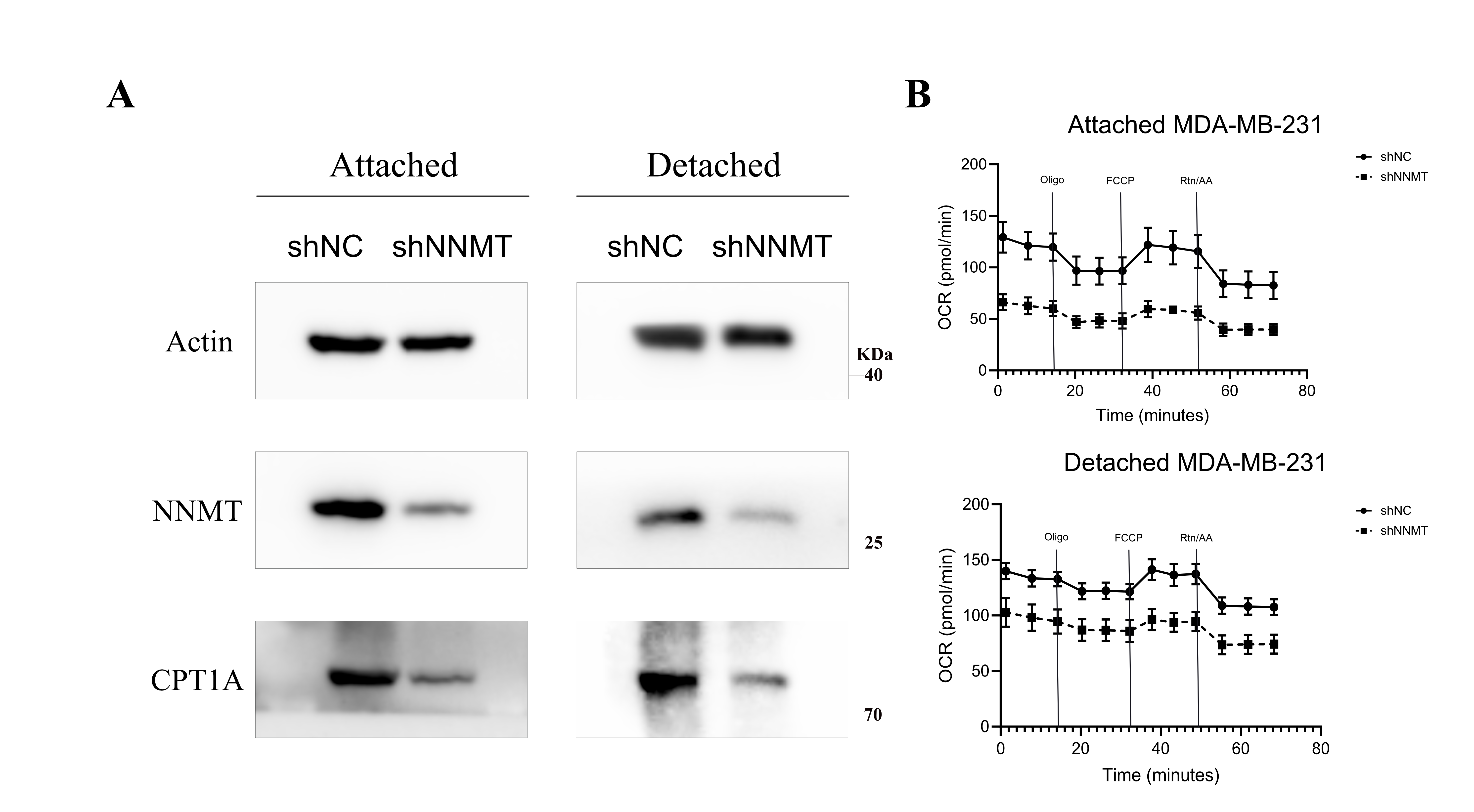


FigS5: NNMT promotes MDA-MB-231 cells FAO both under attachment and detachment. A: WB analysis showed CPT1A was downregulated in NNMT knock-down MDA-MB-231 cells compared to control. B: Measurements of FAO rate in attached and detached MDA-MB-231 cells using the seahorse XF96e extracellular flux analyzer.


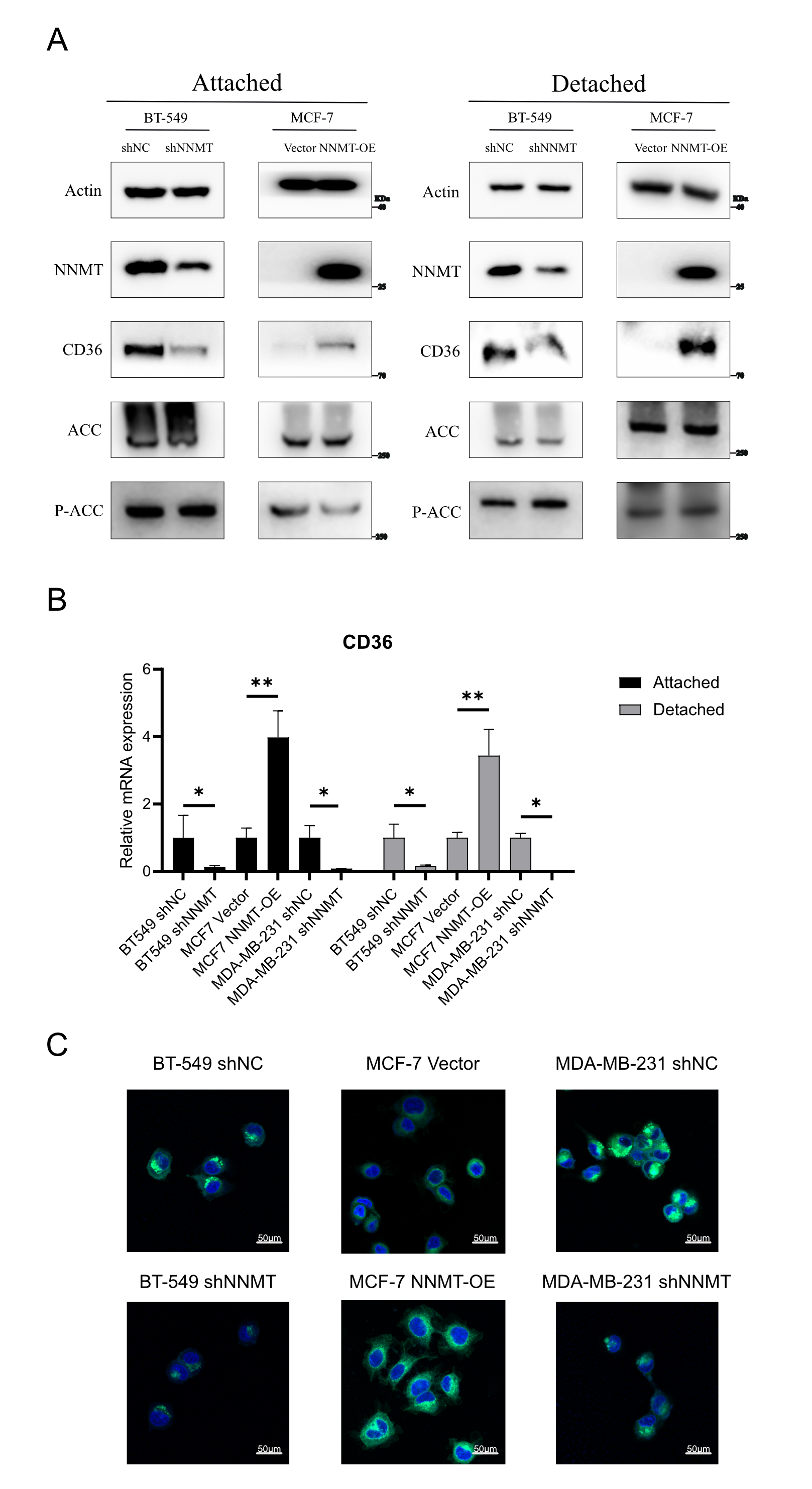


FigS6: NNMT promotes CD36 expression in breast cancer. A: The presentative results of CD36, ACC and P-ACC expression on the cell membrane in MCF-7 and BT-549 cell models by WB. B: QPCR analysis showed CD36 expression was regulated by NNMT in breast cancer cell lines. C: Representative results of BODIPY lipid uptake assay in BT-549, MCF-7, and MDA-MB-231 cell models using confocal microscope. Scale bars, 50μm.


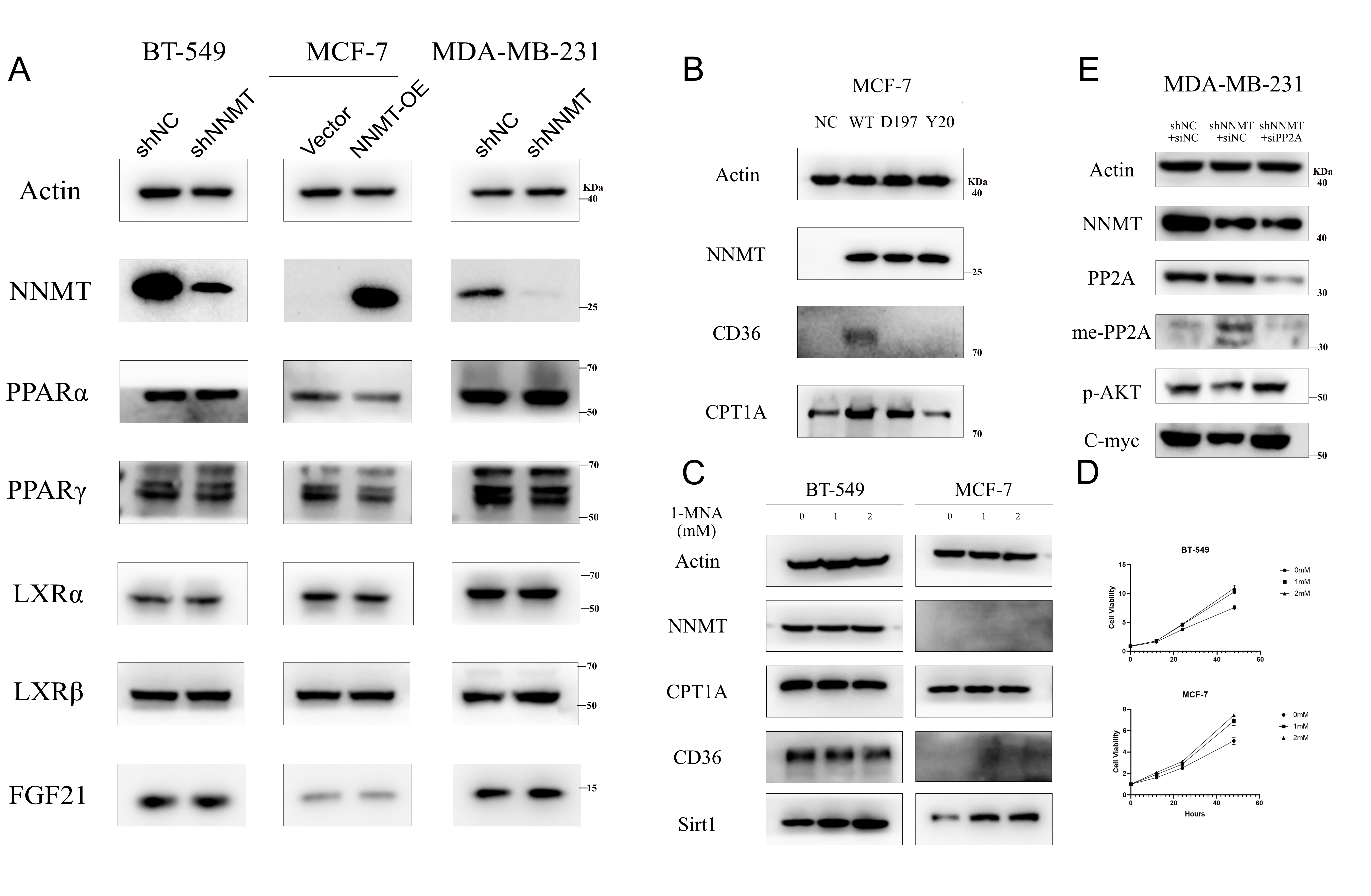


FigS7: NNMT didn’t regulate CPT1A and CD36 through the PPAR pathway but through its N-methyltransferase activity. A: WB analysis showed NNMT did not affect PPARs pathway gene expression such as PPARα, PPARγ, LXRα, LXRβ, and FGF21 in breast cancer. B: WB analysis showed NNMT regulated CPT1A and CD36 by N-methyltransferase activity. C: WB analysis showed stimulation with 1-MNA, the metabolite of NNMT, did not induce a significant increase in CPT1A and CD36, while Sirt1 was upregulated, confirming the functional activity of 1‑MNA in our system. D: CCK‑8 assays demonstrated that 1‑MNA promoted cell growth, confirming the functional activity of 1‑MNA in our system. E: WB analysis showed siPP2A treatment reverses the NNMT knockdown-induced attenuation of P-AKT and C-myc.


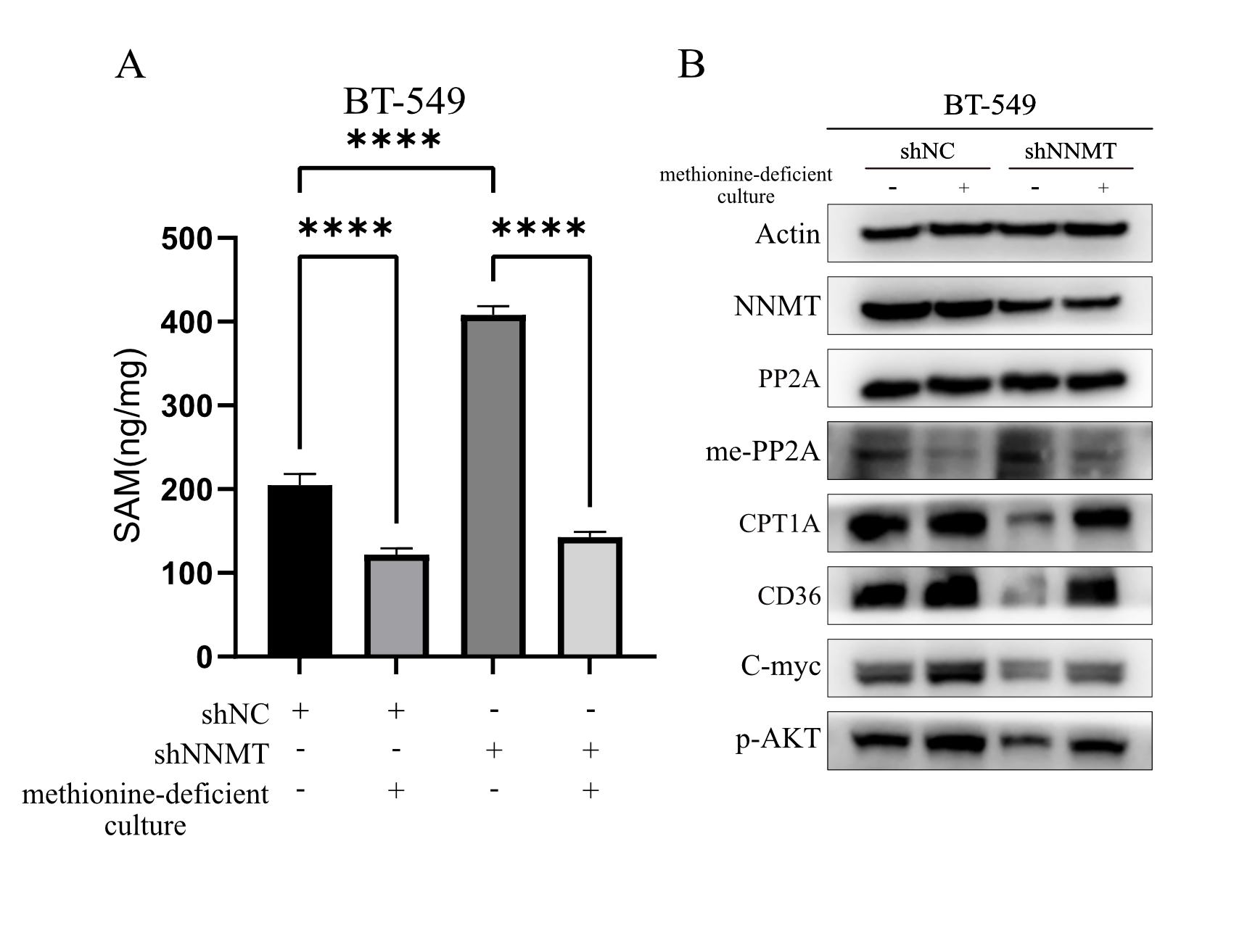


FigS8 A: Intracellular SAM levels were significantly reduced upon treatment with methionine-deficient medium, confirming the effectiveness of the methionine-deprivation condition.B: Western blot analysis indicates that NNMT exerts its regulatory effect on downstream molecules through SAM depletion.


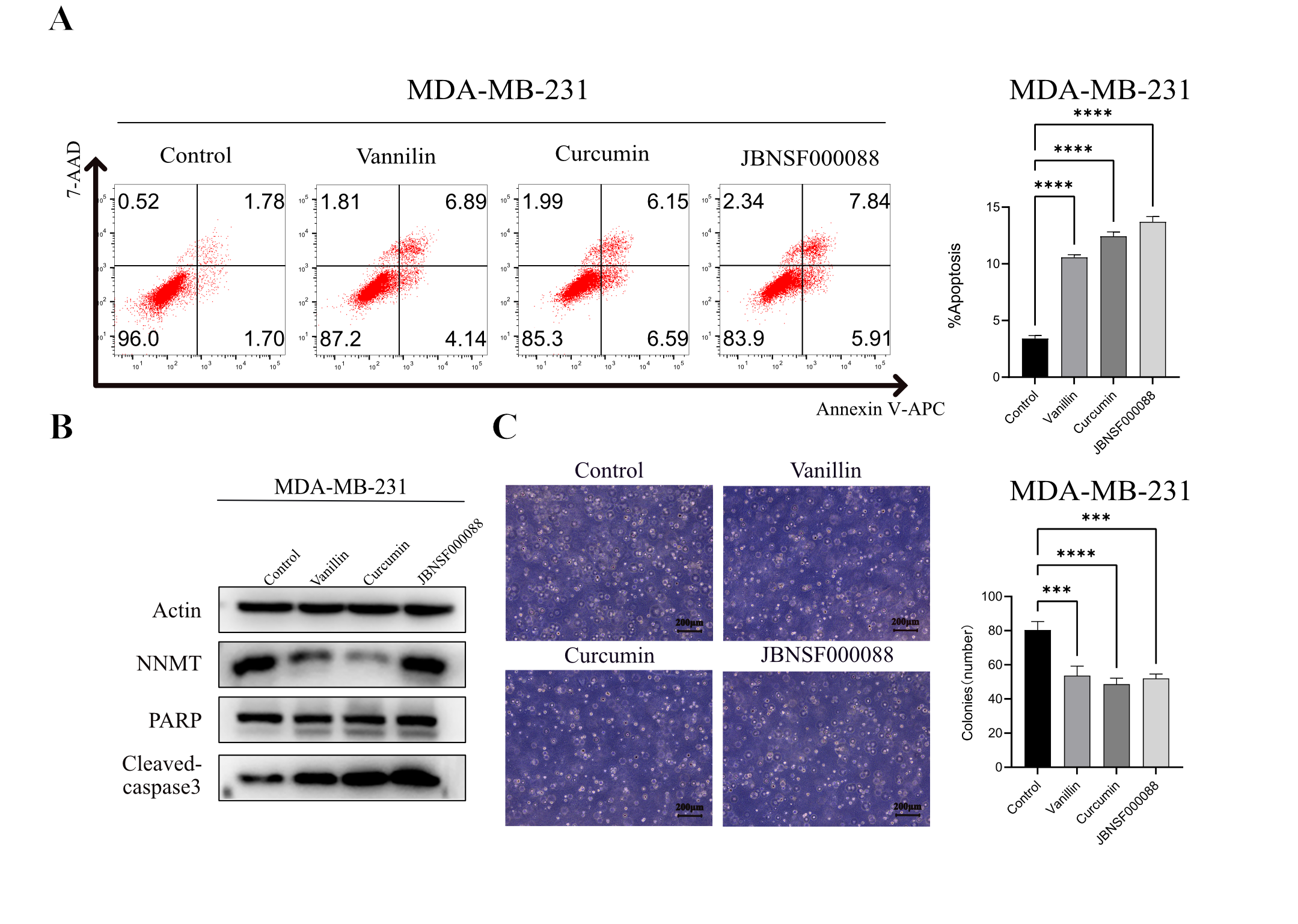


FigS9: Results of Anoikis analysis(A), WB (B), soft agar colony formation assay (C) in MDA-MB-231 cells treated with Vanillin, Curcumin and JBNNSF000088, which significantly inhibited anoikis, indicating the specific role of targeting NNMT in promoting anoikis in breast cancer patient.


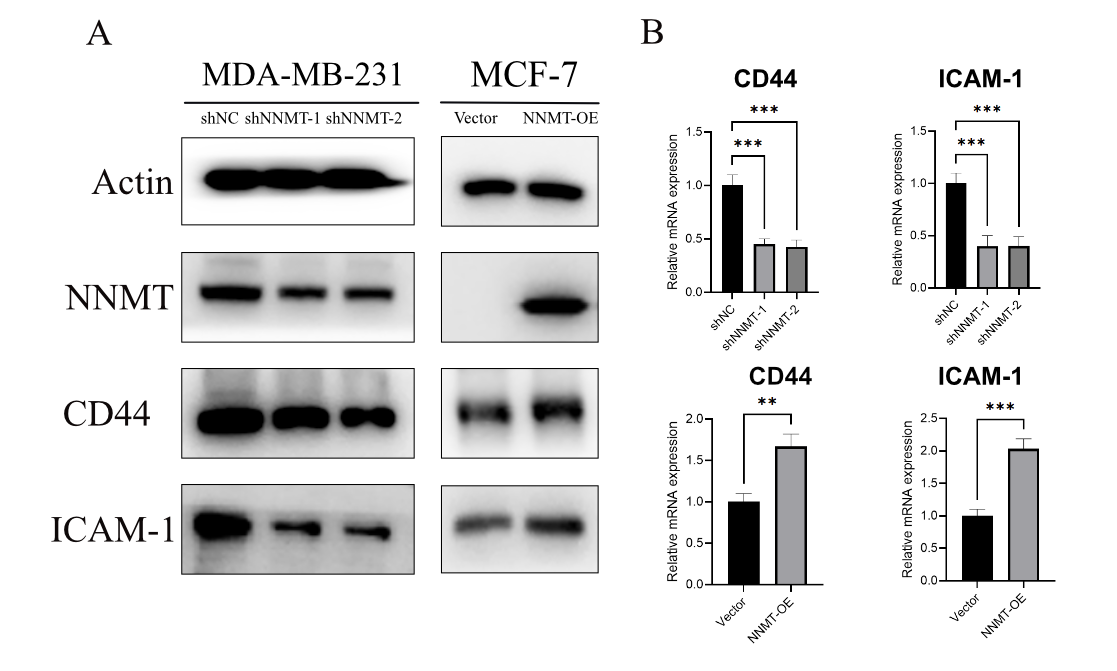


FigS10: A‑B: Western blot and qPCR analyses of MDA‑MB‑231 (A) and MCF7 (B) cell models demonstrate that NNMT regulates the expression of CD44 and ICAM‑1, suggesting its potential involvement in CTC cluster formation.


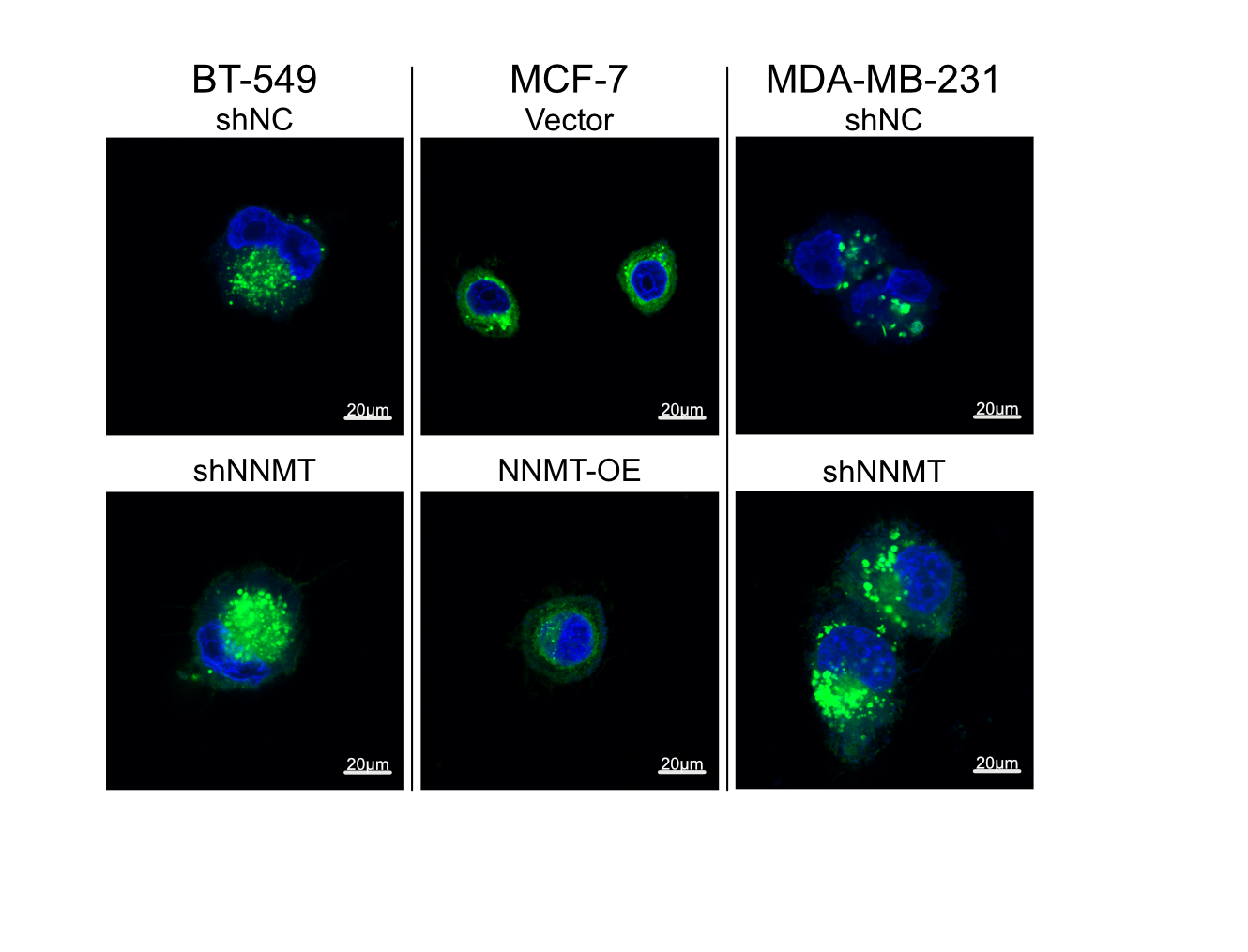


FigS11 Representative results of BODIPY staining in BT-549, MCF-7, and MDA-MB-231 cell models using confocal microscope. Scale bar, 20μm.


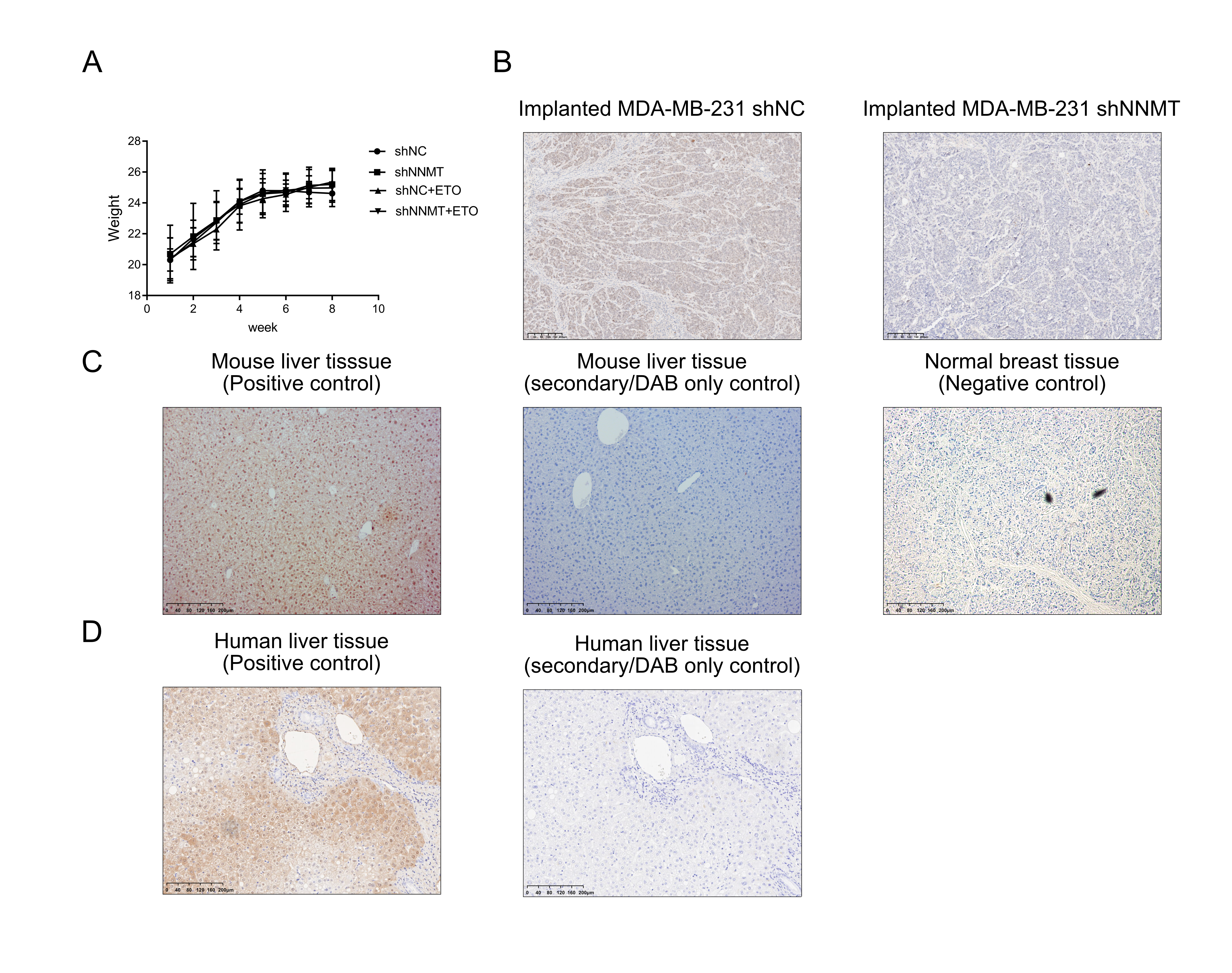


FigS12 The weight growth of the mice model and the image of IHC control. A: The weight growth curve of mice model with etomoxir treatment compared to control. B: Representative result of staining NNMT in MDA-MB-231 shNC and shNNMT primary tumor tissue from CTCs model. Scale bar, 200μm.C: Representative result of staining NNMT in normal breast tissue (negative control) and liver tissue(positive control) by IHC. And the representative result of replacing the NNMT antibody with PBS to stain the same section. Scale bar, 200μm. D: Representative result of staining NNMT in metastatic lung nodules from the E0771 vector group and NNMT-OE group. Scale bar, 100μm.
