# Supplementary material for: Detachment‐Induced FAK‐STAT3‐NNMT Inhibits CTCs Anoikis to Promote Breast Cancer Metastasis by Enhancing Fatty Acid Oxidation
Source: Adv Sci (Weinh). 2026 Mar 12;13(29):e22837. doi: 10.1002/advs.202522837 (PMC13205902; doi:10.1002/advs.202522837)
Supplement: Supplementary file 2 — Supporting File 2: advs74779‐sup‐0002‐Tables1.docx. [file ADVS-13-e22837-s001.docx]

Supplementary Table S1: The clinical characteristics of these patients including age, TNM stage (tumor diameter, lymph node metastasis and distant metastasis) according to the guideline for breast cancer of Chinese society of clinical oncology (Version 2020).

Supplementary Table 2: The results of CTC Detection in blood samples from 45 newly diagnosed breast cancer patients.

Supplementary Table 3: The corrected sequencing results of CTCs versus primary breast cancer tissue source samples according to the combined cohort (GSE209998 and GSE113890).

Supplementary Table 4: The results of the differentially expressed genes in detached MDA-MB-231 cells compared to the attached group.

Supplementary Table 5: The differential gene expression in CTCs compared to primary breast cancer tissue according to the combined cohort (GSE209998 and GSE113890).

Supplementary Table 6: The significantly enriched FAO-related pathways with adjusted P-values (P-adjust < 0.001) in three breast cancer cohorts (GSE4922, GSE9893, and GSE24450).

Supplementary Table 7: Sequences of shNC, shNNMT-1 and shNNMT-2.

Supplementary Table 8: Sequences of primers used in qPCR and ChIP assay.
